# Pilot Randomised Controlled Trial of a Web-Based Intervention to Promote Healthy Eating, Physical Activity and Meaningful Social Connections Compared with Usual Care Control in People of Retirement Age Recruited from Workplaces

**DOI:** 10.1371/journal.pone.0159703

**Published:** 2016-07-29

**Authors:** Jose Lara, Nicola O’Brien, Alan Godfrey, Ben Heaven, Elizabeth H. Evans, Scott Lloyd, Suzanne Moffatt, Paula J. Moynihan, Thomas D. Meyer, Lynn Rochester, Falko F. Sniehotta, Martin White, John C. Mathers

**Affiliations:** 1 Human Nutrition Research Centre, Newcastle University, Newcastle upon Tyne, United Kingdom; 2 Institute of Cellular Medicine, Newcastle University, Newcastle upon Tyne, United Kingdom; 3 Newcastle University Institute for Ageing, Newcastle University, Newcastle upon Tyne, United Kingdom; 4 Department of Applied Sciences, Faculty of Health & Life Sciences, Northumbria University, Newcastle upon Tyne, United Kingdom; 5 Institute of Health and Society, Newcastle University, Newcastle upon Tyne, United Kingdom; 6 Institute of Neuroscience, Newcastle University, Newcastle upon Tyne, United Kingdom; 7 People Services, Redcar & Cleveland Borough Council, Redcar, United Kingdom; 8 Centre for Public Policy and Health, School of Medicine, Pharmacy and Health, Durham University Queen's Campus, Stockton on Tees, United Kingdom; 9 Health and Social Care Institute, School of Health and Social Care, Teesside University, Middlesbrough, Tees Valley, United Kingdom; 10 Fuse, UKCRC Centre for Translational Research in Public Health, Newcastle upon Tyne, United Kingdom; 11 Centre for Oral Health Research, Newcastle University, Newcastle upon Tyne, United Kingdom; 12 Department of Psychiatry & Behavioral Sciences, University of Texas HSC, Houston, Texas, United States of America; 13 Centre for Diet and Activity Research (CEDAR), MRC Epidemiology Unit, University of Cambridge, Cambridge, United Kingdom; 14 Centre for Ageing & Vitality (CAV), Newcastle University, Newcastle upon Tyne, United Kingdom; Indiana University Richard M. Fairbanks School of Public Health, UNITED STATES

## Abstract

**Background:**

Lifestyle interventions delivered during the retirement transition might promote healthier ageing. We report a pilot randomised controlled trial (RCT) of a web-based platform (Living, Eating, Activity and Planning through retirement; LEAP) promoting healthy eating (based on a Mediterranean diet (MD)), physical activity (PA) and meaningful social roles.

**Methods:**

A single blinded, two-arm RCT with individual allocation. Seventy-five adult regular internet users living in Northeast England, within two years of retirement, were recruited via employers and randomised in a 2:1 ratio to receive LEAP or a ‘usual care’ control. Intervention arm participants were provided with a pedometer to encourage self-monitoring of PA goals. Feasibility of the trial design and procedures was established by estimating recruitment and retention rates, and of LEAP from usage data. At baseline and 8-week follow-up, adherence to a MD derived from three 24-hour dietary recalls and seven-day PA by accelerometry were assessed. Healthy ageing outcomes (including measures of physiological function, physical capability, cognition, psychological and social wellbeing) were assessed and acceptability established by compliance with measurement protocols and completion rates. Thematically analysed, semi-structured, qualitative interviews assessed acceptability of the intervention, trial design, procedures and outcome measures.

**Results:**

Seventy participants completed the trial; 48 (96%) participants in the intervention and 22 (88%) in the control arm. Participants had considerable scope for improvement in diet as assessed by MD score. LEAP was visited a median of 11 times (range 1–80) for a mean total time of 2.5 hours (range 5.5 min– 8.3 hours). ‘Moving more‘, ‘eating well’ and ‘being social’ were the most visited modules. At interview, participants reported that diet and PA modules were important and acceptable within the context of healthy ageing. Participants found both trial procedures and outcome assessments acceptable.

**Conclusions:**

The trial procedures and the LEAP intervention proved feasible and acceptable. Effectiveness and cost-effectiveness of LEAP to promote healthy lifestyles warrant evaluation in a definitive RCT.

**Trial Registration:**

ClinicalTrials.gov NCT02136381

## Introduction

An unhealthy diet, inactivity, smoking and a range of environmental factors accelerate the molecular and cellular damage associated with ageing, whereas a healthier diet, frequent physical activity (PA), and not smoking may delay the accumulation of damage and delay or prevent the development of age-related frailty, disability and disease [1].

A wealth of observational data supports the hypothesis that health and wellbeing in later life are influenced strongly by behavioural factors and social conditions [2,3] but there is a dearth of evidence about interventions, particularly those targeting multiple behaviours and social factors [4], that are effective in promoting health and wellbeing during ageing. When longevity is increasing [5] and many governments are introducing policies to extend working life[6], effective lifestyle interventions may offer potential to enhance healthy ageing. Life transitions represent windows of opportunity [7] in which behaviour change interventions may be more effective [8]. The retirement period may be particularly important for healthy ageing since this life transition is often associated with changes in PA [9].

Behavioural interventions to change lifestyle and reduce the burden of age-related disease that have addressed target populations using a one-size-fits-all approach [10], are of limited, or uncertain, long-term effectiveness [11–13] and may widen inequalities [14]. Interventions delivered via digital technologies are used increasingly and may help to overcome such limitations by offering advantages including convenience, scalability, personalisation/stratification, sustainability, and reduced costs [15]. Other limitations of existing evidence include poor study design, absence of a theoretical basis and, importantly, no *a priori* demonstration of the feasibility of the intervention with respect to changing the specific targeted behaviours. Guided by the UK Medical Research Council’s framework for developing and evaluating complex interventions [16,17], we developed a web-based intervention to promote healthy eating, PA, and meaningful social roles in the retirement transition. We designed and built this intervention based on systematic reviews of the literature reporting that interventions with people of retirement age are effective in increasing consumption components of the Mediterranean diet (MD) [11], increasing PA [12] and promoting meaningful social roles [13] and from these we identified intervention components, including behaviour change techniques, appropriate for the target population [18,19]. Furthermore, our qualitative research suggested that lifestyle interventions that address challenges within the retirement transition are acceptable [20] and that personalised approaches to increase PA may be more successful [21]. Using a sequential, iterative approach to intervention development and employing co-design methods, we developed an interactive, modular, web-based lifestyle intervention called LEAP (Living, Eating, Activity and Planning in retirement) [22] which aims to change key lifestyle factors (diet, PA and social connections/roles) and to promote health and wellbeing. The benefit of such interventions needs to be assessed in terms of health gain and we have developed a suite of outcome measures capturing the healthy ageing phenotype (HAP) [23] whose acceptability was evaluated in this study.

We report the results of a pilot randomised controlled trial (RCT) with people of retirement age recruited from workplaces, which tested the feasibility and acceptability of trial design and procedures including: randomisation, recruitment and trial retention; the feasibility and acceptability of LEAP; and the feasibility, acceptability and validity of outcome measures for a future definitive trial.

## Methods

**Fig 1:**
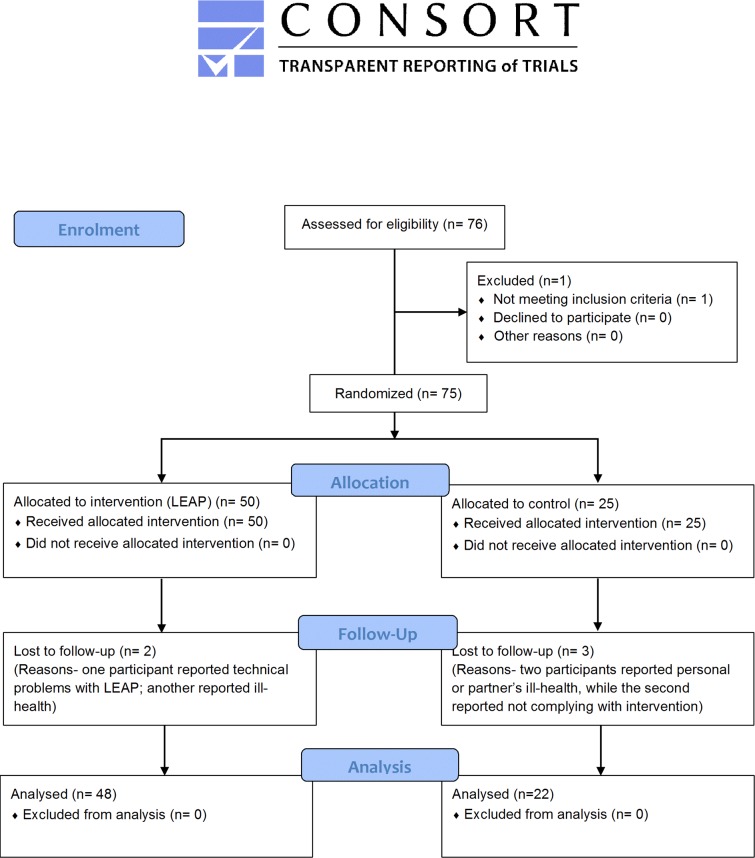
CONSORT 2010 Flow Diagram.

The Newcastle University Faculty of Medical Sciences Ethics Committee approved this study (registration number 00745/2014). No deviations from the protocol occurred (see supporting information file). Written informed consent was obtained for participation and video recording. This study is reported according to the CONSORT [24] (**Fig 1 and Table A in S1 File**) and TIDieR [25] (**Table B in S1 File**) guidelines. The trial was registered at ClinicalTrials.gov (NCT02136381).

### Study design and randomisation

This was a single-blind, individually randomised, controlled parallel two-arm pilot intervention trial undertaken between May and December 2014. Eligible participants were randomised in a 2:1 ratio to either intervention or a ‘usual care’ control arm. This ratio was chosen I order to obtain as much information as possible about LEAP. The randomisation sequence (http://www.randomization.com) was generated by a person unrelated to the project. Because participants were recruited from workplaces, a randomisation list was produced for each workplace to minimise potential confounding. A project administrator contacted participants via email and informed them of their group allocation; during the trial, participants could contact the administrator via email or telephone if technical problems arose relating to LEAP.

Participants were assessed at baseline (before randomisation) and eight weeks later. They attended two 1.5 hour visits at one of three locations across Northeast England for assessments undertaken by researchers blinded to group allocation.

### Participant recruitment strategy

Participants were recruited from relatively large employers covering a range of occupations in Northeast England. Health Improvement Specialists—involved in workplace health activities via the North East Better Health at Work award [26]—facilitated contact with employers who in turn supported participant recruitment. Access to the number of eligible individuals within each employer was not available. The human resources and health and safety departments in companies that agreed to participate were responsible for advertising the research project to employees. Potential participants contacted the research team to receive study information and to arrange a telephone appointment to assess eligibility. No participant was declined participation and once the sample size proposed was achieved recruitment was closed.

### Inclusion/exclusion criteria

To be eligible, participants had to have retired in the last two years, or planned to retire from full-time work in the next two years. Participants were required to have access to a personal computer (PC), tablet or smartphone, and to have reliable internet access at home, work and/or via mobile networks. Participants were excluded if they could not comprehend and speak English fluently or had severe mental health conditions that could affect compliance, as assessed by a brief screening questionnaire developed for this study. To facilitate compliance with interventions, data from participants scoring ≥20 on the Centre for Epidemiologic Studies Depression Scale (CESD) [27], and/or scoring two or more standard deviations below the mean for age, gender, and education on the Paired Associate Learning (CANTAB) [28], were excluded. In addition, participants with raised blood pressure (>180mmHg/>110mmHg), identified during the first health assessment session, were advised to consult their physician as soon as possible and participants concerned about participating due to a health condition were advised to consult their physician about their ability to participate.

### Interventions

The intervention was based on the Health Action Process Approach, a theoretical framework recognising the importance of planning, self-efficacy and self-regulatory strategies (action control) for behaviour change [29].

#### LEAP intervention arm

We developed LEAP, a web-based intervention that can be accessed on a PC, tablet or mobile phone, for this project [22]. The intervention comprises the following five modules: 1) Time, 2) Changing Work, 3) Moving More, 4) Being Social and 5) Eating Well, as well as a diary and a dashboard section to assist with site navigation. The intervention content was personalised to the participant based on information provided during use. Following randomisation to LEAP, participants were instructed, via email, to access the website (http://demo.leapsuite.co.uk/) and register.

A brief description of the intervention modules is given below and a detailed description in supplementary material (**Box A** in **S1 File**).

The ‘Time’ module encouraged users to reflect on how they spend their time currently and whether and how they would like to change time use later in their retirement transition, focusing on time spent working, caring for others, pursuing hobbies, being physically and socially active, doing household duties and having undefined ‘free time’.

The ‘Moving More’ module supported users to be more physically active and to sit less, and encouraged them to self-monitor their steps daily using a dual-axis pedometer (Omron HJ203). The module invited the user to set PA and step goals and to return to the module regularly to update their step count, review goals, schedule activities, and consider the barriers and solutions to being more physically active.

The ‘Being Social’ module explored the potential benefits of having a meaningful occupation or other role (e.g. volunteering) and spending time with friends, family and work colleagues. The user was prompted to explore significant social relationships, consider how these might change through the retirement transition, and to add further relationship ‘types’ (e.g. to make new friends). The tool presented a list of potential social activities for each relationship type, which could be filtered based on cost, accessibility (e.g. for people with limited mobility), and intensity (PA level).

The ‘Eating Well’ module encouraged the user to consider their current diet and to make changes towards a more Mediterranean-style eating pattern (MD) [30]. Users completed a 14-item validated questionnaire assessing MD adherence [31], and received brief feedback tailored to diet features on which they scored sub-optimally. Users received ideas for snacks, drinks, breakfast, light meals and main meals to help put the feedback into practice. Users were prompted to reflect on the goals they would like to achieve by eating better, and self-generated solutions to overcome barriers to improved eating patterns.

The ‘diary’ section presented the user’s scheduled activities for the next two weeks. This information could be downloaded to the user’s computer calendar or printed.

The ‘dashboard’ section presented a summary of the activities, tools and resources with which the user had engaged or had saved to view later. It prompted the user to revisit modules, to report on the activities in which they had engaged, to revise their goals and to schedule new activities.

#### Control arm

Participants randomised to the control arm were instructed, via email, to use the NHS Choices website (http://www.nhs.uk/LiveWell/Pages/Livewellhub.aspx), provided by the UK Department of Health, which served as the ‘usual care’ comparator. NHS Choices provides a comprehensive health information service which encourages people to make healthy choices about smoking, drinking, eating and exercise and provides information on finding and using NHS services. Participants were encouraged to access the health resources and information on the pages labelled as men’s health 40–60, men’s health 60-plus, women’s health 40–60, women’s health 60-plus, as appropriate.

### Study outcomes

The primary aims of this study were to establish the feasibility and acceptability of the intervention, the trial design and procedures, and the outcome measures for a definitive trial and so we focussed on recruitment and retention rates, experiences of participants during the trial, usage of LEAP, and compliance with, and variability (mean and 95% CI) of, outcome measures.

Feasibility of the trial design and procedures included recruitment and retention rates, and qualitative feedback on randomisation. Feasibility of LEAP was evaluated by usage data and qualitative feedback from participants. Web analytics tracked usage of LEAP using PiWik (http://piwik.org/) and Google analytics (http://www.google.com/analytics/) software to summarise data at individual participant and aggregated group levels, respectively. The number of visits to LEAP and to each webpage within LEAP, time spent using LEAP and average duration of each visit, and number of activities scheduled by participants during the trial were calculated.

Dietary intake was assessed using the multiple pass 24-hour recall method[32] and used to evaluate MD adherence (assessed using the 14 items scoring system [31]). At baseline and follow-up, participants completed 24-hour dietary recalls on three non-consecutive days, including two weekdays and one weekend day, via the Oxford WebQ [32]. To minimise between-day variability, participants completed dietary recalls on the same days of the week at each time-point. To test the veracity of energy intakes (EI), the EI to Basal Metabolic Rate (BMR) Ratio (EI/BMR) was calculated [33]. BMR was estimated using the sex-specific Henry equations[34]. The diet-related outcomes were acceptability of the assessment procedures and MD adherence before and after the intervention.

To measure PA, participants wore a low cost tri-axial accelerometer (Axivity AX3, York, UK; dimensions: 2.3×3.3×0.8 cm, weight 9g: sampling frequency 100-Hz, resolution: 16-bit, range: ±8g) on the skin at the fifth lumbar vertebra for 7-days at baseline and at follow-up. Ambulatory (stepping/walking) bouts were extracted using a bespoke MATLAB programme with a threshold (60s) applied to the data to account for more purposeful walking[35]. Characteristics representing volume, pattern (alpha) and variability (*S*_*2*_) of ambulatory activity bouts (periods of time spent walking) were established as reported previously[36]. Volume described the number of steps per day, the number of bouts per day, ambulatory time per day (minutes) and the longest bout spent in walking during the assessment week (minutes). Alpha (α) describes the distribution of ambulatory bouts according to time and is described by the power law distribution exponent. A lower α indicates that a participant accumulated ambulatory time with a larger proportion of long ambulatory bouts >10 minutes[37]. Variability (*S*_*2*_) was used to represent the ‘within subject’ variability of bout length and was calculated using a maximum likelihood technique[38].

Feasibility and acceptability of PA outcome measures were established by assessment completion rates and qualitative feedback (see below). A battery of measurements of the HAP[23,39,40] including cognition, physical capability, physiological, and psycho-social wellbeing were collected, and details of its acceptability are reported here; however the measures themselves will be reported in a spate paper elsewhere.

The socioeconomic status of participants was assessed via the Index of Multiple Deprivation (IMD) using the participants’ home postcodes with each participant neighbourhood defined using Lower Layer Super Output Areas (LSOA) [41].

### Data management and quantitative analysis

All statistical analyses were undertaken using IBM SPSS Statistics version 22 for Windows. Distributions of continuous variables were tested for normality using the Shapiro-Wilk test. Since data were normally distributed, results are presented as means and 95% CI.

### Qualitative interviews and analysis

Semi-structured face-to-face interviews were undertaken (at the same venues used for baseline and follow-up data collection) with a sub-sample of participants (N = 14; 10 LEAP intervention, 4 control) exploring reasons for participation or non-participation, experiences of participation, using the LEAP modules, acceptability of LEAP and outcome measures assessments. Interviews lasted from 20 to 60 minutes and were longer for intervention arm participants. Interviews were audio-recorded and the recordings transcribed verbatim. Thematic analysis was conducted [42] identifying key themes, which were refined until data saturation was reached[43].

## Results

### Recruitment and characteristics of participants

Recruitment from workplaces was a successful approach. Employers comprised two large supermarkets and depots, a public transport company, a petrochemical manufacturing company, the UK’s tax and customs authority, and three local government authorities. In total 75 adults were recruited and their demographic and anthropometric characteristics are shown in **Table 1**.

**Table 1:** Characteristics of study participants at baseline.

	Control (n = 25)	Intervention (LEAP) (n = 50)
**Age (yrs)** [Mean ± SD (95% CI)]	62.0 ± 3.9 (60.6 to 63.4)	60.9 ± 3.4 (59.9 to 61.9)
**Height (cm)** [Mean ± SD (95% CI)]	164.8 ± 7.6 (161.3 to 168.2)	165.9 ± 9.2 (163.5 to 168.4)
BMR* **MJ/day** [Mean ± SD (95% CI)]	5.9 ± 0.9 (5.6 to 6.3)	5.9 ± 1.1 (5.6 to 6.2)
**Index of Multiple Deprivation**		
Mean ± SD (95% CI)	5.0 ± 2.9 (3.8 to 6.2)	5.8±2.4 (4.5 to 6.5)
Within the 50% most deprived (n(%))	14 (56)	24 (48)
Within the 50% least deprived (n(%))	11 (44)	26 (52)
**Sex** [Number (%)]		
Male	6 (24)	12 (24)
Female	19 (76)	38 (76)
**Marital Status** [Number (%)]		
Married	20 (80)	43 (86)
Single	1 (4)	1 (2)
Divorced	2 (8)	4 (8)
Widowed	2 (8)	2 (4)
**Smoking habits** [Number (%)]		
Never	12 (48)	33 (66)
Ex-smoker	13 (52)	14 (28)
Current Smoker	0 (0)	3 (6)
**Retirement status** [Number (%)]		
Retired	1 (4)	2 (4)
Pre-retirement	24 (96)	48 (96)

* BMR = Basal Metablic Rate estimated using the Oxford equations by Henry et al. (34)

Participants were mostly women (ratio women: men, 3:1), aged 61±4 years, overweight (BMI 27±5 kg/m^2^), mostly married (84%) and non-smokers (95%). Participants randomly allocated to the Control and to LEAP arms were well-matched for age, anthropometry, gender, IMD, marital status and smoking habits (**Table 1**).

### Study completion

High rates of study completion were observed with 70 participants (93% of those randomised) returning for the 8-week follow-up assessment [48 (96%) participants in the LEAP and 22 (88%) in the control arm]. The willingness of participants to be recruited and take part in tests demonstrated the feasibility of the pilot design.

### Use of the LEAP intervention platform

Forty-eight of the 50 participants randomised to LEAP used the platform at least once over the 8-week trial period. Mean number of visits per user was 11.4 (SD = 12.7; range 1–80) for a mean total time spent of 2.56 hours (SD = 1.94, range 5.5 min–8.3 hours); mean length of each visit was 17.9 minutes (SD = 10.4; range 5.5–47 min). After the end of the trial, 17 users (35%) continued using LEAP. The ‘moving more’, ‘being social’ and ‘eating well’ modules were the most visited LEAP modules. Reviewing goals, meal ideas, and exploring social roles were the pages most often used. The diary feature on LEAP was used by 30 of 48 users, who scheduled an average of 22 (range 1–94) activities over the 8-week trial period. On average there were 14 ‘moving more’ activities, 5 ‘eating well’ meals and 14 ‘being social’ activities scheduled per user. Half of all scheduled activities included where, and with whom, the activity would be undertaken.

### Qualitative outcomes

Fourteen participants, seven men and seven women, mean age 62.6 years (range 58–71), three retired and eleven about to retire, were interviewed.

#### Exploring reasons for participation or non-participation in the study

Participants described ‘full’ and ‘active’ lives, loneliness was considered more an issue of ‘later life’ and participating in research was an example of ‘actively pursuing interests’ and ‘getting involved’.

*“There was loads of different things … that you could look at and then take the link to a local organisation … and try it [e*.*g*. *PA activities] … certainly [there are resources that] if you had to go and locate it yourself you’d probably give up or not bother*, *but the fact the website’s got it and it’s probably got suggestions that you’ve … not even thought of … you … would use them*. *…*.*”*. [ID004, Male, LEAP, aged 60, occupational pension, working part-time]

#### Intervention website (LEAP)

Regarding the use of LEAP, two themes emerged. First, conceptual relevance; LEAP modules were regarded as addressing key concerns through retirement transitions:

*“The idea is good*, *the fact that you can look at what you’re spending now and what you’re budgeting now and then see how that compares into retirement because … the two things you worry about are your health and your finances and both have to come together to have a happy retirement*. *So*, *being able to show a plan of now and then is handy and I think as well some of the links were good … the government website [with pensions advice]”*. [ID004, Male, LEAP, aged 60, occupational pension, working part-time].

Changing needs as retirement progressed were highlighted and LEAP’s usefulness in this regard was discussed:

*“At the moment we want to retire … travel or … make the most of time together*, *a year down the line you might say well you want to take part in some voluntary work … and it [LEAP] will be handy as a way to go and look to see where opportunities are … So I think [there are] lots of things that you could dip into”*. [ID0004, Male, LEAP, aged 60, occupational pension, working part-time].

Second, design, navigation and technical issues such as understanding ‘how the site worked’ were problematic for a few users. In most cases, these were minor issues but some described them as intensely frustrating.

Diet and PA modules were regarded as important and entirely acceptable within the context of healthy ageing. The use of the pedometer was described as a ‘good motivator’. Actively seeking help via the social roles and activities domain appeared to be less acceptable, unless embedded in other activities:

*“I … [don’t] want to go on … organised [walks] … the [walkers are] dressed up in [uniforms with slogans like] ‘WALK LEADER!’… I’m not going to have anything to do with that’! But … I go [walking] by myself with the dog [after being prompted by the website], and its marvellous how with the dog gets you into conversation … if I’ve got the dog with me everyone replies and you get to meet other dog walkers …. So it does have a beneficial effect.”* [ID026, Male, LEAP, aged 71, occupational pension, working part-time]

The content and format of LEAP, which could be personalised, were acceptable and reinforced the need for an intervention taking account of the heterogeneity of adults in the retirement transition.

### Social connections/ roles

Prompting about social roles and activities was regarded as important, particularly for those individuals who had not given much thought to how they would structure their retirement:

Interviewer: *Was there anything … that attracted you in terms of thinking through … work exits or what you might want to do in retirement?*

Respondent: “*[…] It was really more … health*. *Because I have quite a number of interests so I’m not particularly concerned about how I’m going to fill my time [but] it has kick-started me and I have enrolled in a quilting course* [ID011, Female, LEAP, aged 62, occupational pension, working part-time]

Respondent: *“I didn’t have much of a plan about retirement … it really was like a cut-off […] I’d still go and see friends … but I felt as though I didn’t have much of a meaning once I’d finished my job … I didn’t feel I was doing anything of worth*. *… so the website*, *the whole idea of doing something active*, *… was really picking us up a bit*. *… suggested lots of ways*, *avenues*, *particularly for helping people [e*.*g*. *volunteer work]*, *but I haven’t pursued those yet*. *I’m not sure why*. *It’s committing yourself really*.*”* [ID022, Male, LEAP, aged 65, occupational pension, retired]

Whilst the social roles module was described as appealing, some participants avoided an ‘older adult’ identity and felt that social support tools were less useful to them.

*“… [on the website] one [module] is ‘how to make friends’ … well I’m not interested in that … no*, *the only thing I would like to do is control my weight*.*”* [ID026, Male, LEAP, aged 71, occupational pension, working part-time]

### Outcome measures

The HAP assessment took approximately 1.5 hours. Participants found the measurements acceptable and, for many, it was an enjoyable experience. Interestingly, this assessment met the expressed ‘needs’ and expectations of some participants (i.e. *“to receive a health check”*), and face-to-face contact with members of the research team was valued.

### Proposed outcomes for a definitive trial

#### Food consumption measures

Completers of the trial (93% of those randomised) undertook, and completed successfully, the online 24-hour dietary recalls at baseline and at follow-up. No problems were reported in completing the dietary recalls on the required days and the online tool was described as user-friendly.

Baseline mean energy intake was 8.9±3.1 MJ/d which represented an EI/BMR ratio of 1.5 (**Table 2**). Mean daily energy intakes at the end of the intervention were 33 and 422 kJ/d lower (for Control and LEAP, respectively) than at baseline (**Table C in** S1 File) which accords with the small reductions in body weight (-0.3 vs -0.6 kg) and waist circumference (-0.4 vs -0.9 cm) observed for the control and intervention arms, respectively (**Table 2**).

**Table 2:** Anthropometric status at baseline and follow-up (8 weeks).

	Control (n = 22)		Intervention (LEAP) (n = 48)	
	Baseline	Follow-up	Baseline	Follow-up
	Mean (95% CI)	Mean (95% CI)	Mean (95% CI)	Mean (95% CI)
**Weight (kg)**	73.9 (67.7 to 80.2)	73.6 (67.5 to 79.8)	74.2 (69.8 to 78.7)	73.6 (69.2 to 78.0)
**Body mass index (BMI) (kg/m**^**2**^**)**	27.3 (25.4 to 29.3)	27.2 (25.2 to 29.1)	26.8 (25.4 to 28.2)	26.6 (25.2 to 28.0)
**Waist Circumference (cm)**	92.3 (87.2 to 97.4)	91.9 (87.1 to 96.8)	90.4 (86.8 to 94.1)	89.5 (85.9 to 93.0)
Body fat mass (kg)*	25.6 (21.8 to 29.4)	25.4 (21.6 to 29.2)	24.7 (22.0 to 27.3)	24.5 (21.9 to 27.2)
Fat-free mass (kg)*	47.0 (42.3 to 51.7)	48.3 (44.2 to 52.4)	49.5 (46.3 to 52.8)	48.5 (45.7 to 51.3)
Total body water (kg)*	34.2 (31.0 to 37.4)	33.7 (30.7 to 36.7)	34.9 (32.7 to 37.1)	34.0 (31.9 to 36.1)

* Estimated using bioelectrical impedance.

At baseline, mean consumption of pulses, nuts, fish, and fruit and vegetables (4.1 vs 4.8 portions/day for the control and LEAP arms, respectively) was generally low. Both arms reported consuming olive oil, with relatively higher frequency and intake in the LEAP arm. MD scores at baseline were comparable across study arms and relatively low (**Table 3**).

**Table 3:** Self-reported food intake at baseline and at follow-up (8 weeks).

	Control (n = 22)		Intervention (LEAP) (n = 48)	
Food group (portion/day)	Baseline	Follow-up	Baseline	Follow-up
	Mean (95% CI)	Mean (95% CI)	Mean (95% CI)	Mean (95% CI)
**Fruits**	2.0 (1.4 to 2.7)	1.9 (1.2 to 2.5)	2.2 (1.8 to 2.7)	2.2 (1.7 to 2.6)
**Vegetables**	2.1 (1.3 to 2.9)	2.0 (1.3 to 2.8)	2.6 (1.9 to 3.1)	2.4 (1.9 to 3.0)
**Meat**	0.9 (0.6 to 1.3)	0.7 (0.4 to 0.9)	0.8 (0.5 to 1.0)	0.7 (0.4 to 0.9)
**Carbonated drinks**	0.1 (0.0 to 0.3)	0.1 (0.0 to 0.2)	0.3 (0.1 to 0.4)	0.2 (0.1 to 0.4)
**Ratio of Poultry to Red meat**	0.3 (0.0 to 0.6)	0.4 (-0.2 to 1.0)	0.5 (0.3 to 0.8)	0.6 (0.0 to 1.2)
**Pulses**	0.1 (0.0 to 0.2)	0.1 (0.0 to 0.2)	0.1 (0.1 to 0.2)	0.1 (0.1 to 0.2)
**Fish**	0.2 (0.0 to 0.3)	0.4 (0.2 to 0.5)	0.3 (0.2 to 0.4)	0.3 (0.2 to 0.4)
**Nuts**	0.2 (0.0 to 0.3)	0.3 (0.1 to 0.5)	0.2 (0.1 to 0.3)	0.3 (0.1 to 0.4)
**Tomato Sauce**	0.1 (0.0 to 0.1)	0.0 (0.0 to 0.0)	0.0 (0.0 to 0.1)	0.1 (0.1 to 0.1)
**Butter**	0.9 (0.3 to 1.4)	1.2 (0.7 to 1.7)	0.8 (0.5 to 1.2)	0.7 (0.4 to 1.0)
**Desserts**	1.6 (1.2 to 2.1)	1.3 (0.8 to 1.4)	1.1 (0.8 to 1.4)	0.9 (0.7 to 1.1)
**Olive oil**	0.3 (0.1 to 0.4)	0.2 (0.1 to 0.5)	0.4 (0.3 to 0.5)	0.4 (0.3 to 0.5)
**Wine**	0.4 (0.2 to 0.5)	0.3 (0.1 to 0.4)	0.3 (0.2 to 0.4)	0.3 (0.2 to 0.4)
**Olive oil use (Yes/No)**	11/11	8/14	21/27	21/27
**Mediterranean diet score**	3.8 (3.2 to 4.5)	3.8 (3.1 to 4.5)	4.7 (4.2 to 5.2)	4.6 (4.1 to 5.1)

Food groups based on the Mediterranean diet score (maximum score = 14) developed for the PREDIMED study [31]

#### Physical activity measures

Complete accelerometry data at baseline and follow-up were obtained from 56 participants (80% of completers). Participants did not report any problems with wearing the device for 7 days and missing data were due mainly to instrument malfunction (n = 4) and incomplete recordings (n = 10). For all PA measures, outcomes were similar for both treatment groups. **Table 4** shows values for alpha (α), the variability outcome, number of steps and the longest activity bout time (minutes) by intervention arm at baseline and after 8 weeks.

**Table 4:** Parameters of ambulatory activity (assessed using accelerometry) at baseline and at follow-up (8 weeks).

	Control		Intervention (LEAP)	
	Baseline	Follow-up	Baseline	Follow-up
	Mean (95% CI)	Mean (95% CI)	Mean (95% CI)	Mean (95% CI)
Alpha (α) *	2.49 (2.39 to 2.59)	2.58 (2.49 to 2.67)	2.49 (2.40 to 2.56)	2.52 (2.42 to 2.62)
Variability ** (seconds within activity bout variability)	0.61 (0.54 to 0.68)	0.59 (0.52 to 0.65)	0.62 (0.57 to 0.67)	0.61 (0.56 to 0.66)
Steps (number per day)	7691 (4586 to 10795)	7181 (3978 to 10384)	7056 (4986 to 9126)	6916 (4909 to 8922)
Number of activity bouts per day	31 (17 to 45)	32 (16 to 48)	28 (20 to 36)	29 (21 to 37)
Ambulatory time per day (minutes)	88 (36 to 140)	82 (28 to 167)	81 (45 to 117)	80 (46 to 114)
Longest activity bout spent in walking during the assessment week (seconds)	1382 (91 to 2856)	1231 (48 to 2413)	1302 (473 to 2131)	1278 (292 to 2264)

*Values were calculated using 60s threshold [36]. Alpha describes the distribution of ambulatory bouts according to their time and is described by the power law distribution exponent. A lower α indicates that a participant tends to accumulate ambulatory time with a larger proportion of long ambulatory bouts.

** Values were calculated using 60s threshold [36]. Variability was used to represent the ‘within subject’ variability of bout length. This was calculated using a maximum likelihood technique.

## Discussion

### Principal findings

The evaluation indicated a high level of acceptability of the study design, a high study completion rate, acceptable levels of compliance with the intervention including high usage of the LEAP platform, high completion rates for data collection, as well as confirmed feasibility and acceptability of the outcome assessment procedures. Acceptability of the intervention was also high. Overall participants reported that the LEAP domains of ‘eating well’, ‘moving more’ and ‘being social’ were important for their health and wellbeing in retirement, a finding which is supported by data on the most accessed components of LEAP. Our results are in line with studies among Dutch adults indicating that eHealth lifestyle modules focusing on preventive behaviours, such as PA and diet, are preferred by participants [44–46].

In agreement with previous findings [47], participants reported liking the use of a pedometer to facilitate self-monitoring of progress towards PA goals. There was also good acceptability of, and participants enjoyed undertaking, the battery of tests used to characterise the HAP [23,39,40]. Most participants reported having good relationships and ‘full lives’. Admitting concerns with social relationships and sense-of-purpose may be perceived as not ‘managing life’ very well. Some evidence indicated that ‘social’ aspects were important but more acceptable when embedded in other activities (e.g. social elements of PA). This suggests that a social relationship tool may be more useful if embedded within the eating well and PA modules. The intervention targeted participants in the retirement transition and our aim of recruiting via employers was successful in attracting participants from the appropriate life-stage. Our randomisation procedures worked well in ensuring good balance between intervention and control arms, and randomisation within workplaces minimised possible socioeconomic confounding as shown by the balance of participants living most and least deprived areas.

### Strengths and limitations

To our knowledge, this is the first study to pilot a multicomponent, digital intervention to promote health and wellbeing in the retirement transition. Loss to follow-up was low with high rates of study completion (93%) and complete data collection, in contrast with reports from some other trials using digital interventions [48,49]. The use of web-analytics to track use of the LEAP intervention provided objective evidence of the nature and extent of interaction with LEAP. Other strengths include the use of an objective method to assess PA patterns over 7-day periods. Dietary intake was assessed using a validated online 24-hr recall tool [32] and care was taken to avoid potential bias in respect of days of the week of dietary recording. The well-recognized limitations of widely used dietary reporting tools may be amplified when attempting to assess responses to interventions [50] because, by necessity, study participants are aware of the expected dietary behaviour and because repeated measurements are burdensome, which may introduce reporting bias [50]. However energy intakes in this study were higher than the national average for the participants’ age group (energy intakes of 8.88 and 6.78 MJ for men and women respectively in the National Diet and Nutrition Survey [51]) and represented an EI/BMR ratio of 1.5 suggesting little evidence of dietary under-reporting [52].

Relatively small proportions of men and of current smokers took part in the study, which may reduce the generalizability of results [53]. These groups have been identified as less likely to participate in health promotion programmes [54–56] and measures to increase their participation should be employed in a definitive trial. Targeting employers with higher proportions of male employees and using more gender sensitive recruitment strategies might help [57]. Whether this approach would work with small to medium sized companies needs investigation. Other limitations include i) we did not employ a quantitative measure of social roles, ii) the HAP assessment may have had a positive impact on behaviour change and so reduced the potential difference between control and intervention groups, and iii) we acknowledge that there might be heterogeneity introduced by retirement status, however in this study we adopted a pragmatic decision in which we prioritised the recruitment of people around retirement. In a future larger trial we will need to stratify our sample to account for these effects.

### Implications of findings and comparison with other studies

The results of this study are relevant to the development of future web-based lifestyle interventions targeting people of retirement age. This study generated user feedback to fine-tune the LEAP intervention. The feasibility and acceptability of the trial procedures, the intervention and recruitment of people in the retirement transition via workplaces were demonstrated successfully, indicate that these can be optimised and that a definitive trial is warranted [58]. In addition this pilot study provided data on the variability of the main outcomes enabling estimation of the sample size for a definitive trial.

This study adds to the scant literature on the development and implementation of lifestyle-related interventions targeted at people in the retirement transition period. To our knowledge only one previous trial used a computer-based programme to target health behaviours among Dutch retirees; a non-significant trend towards weight loss after 12 and 24 months was reported[59]. Another study on Dutch adults compared the effect of interventions targeting a single behaviour (sequential approach) versus those targeting several behaviours (simultaneous approach); positive, but small, behavioural changes with both approaches were reported [60].

Participants visited LEAP on average 11 times in 8 weeks, a greater frequency than originally predicted (weekly visits). Other studies using internet delivered interventions report similar, or less, use e.g. a study promoting the MD among young Scottish women reported mean 16 visits over 6 months [61].

Web-based interventions are used increasingly and offer advantages including convenience, privacy, scalability, personalisation/stratification, sustainability, and reduced costs [15]. This approach to intervention delivery is becoming more attractive where internet usage e.g. the European Union is high [62]). However, although the use of PCs and of the internet by middle-aged people has increased rapidly in recent years, barriers may limit the use of digital platforms by a minority [63] which will require the design of more tailored, and user-friendly, intervention platforms.

Previous evaluations of workplace interventions aiming to improve individual lifestyles indicate that these could be cost-effective in improving health and reducing sickness-absence from work [26]. Digital lifestyle interventions can provide individualised behaviour change information at low cost and the implementation, and efficacy, of such interventions in real-life settings require testing at scale.

### Conclusions

The findings from this pilot study suggest that people in the retirement transition are motivated to participate in an internet-delivered intervention to improve diet and PA and to strengthen social roles/social connections, lifestyle changes which are expected to enhance healthy ageing. In addition, we found that the web-based LEAP intervention was acceptable and that the study protocols (including those for recruitment and for assessment of behavioural and HAP outcomes) were feasible and acceptable. Findings from this feasibility study will inform further refinement of LEAP and of the trial procedures in preparation for a definitive RCT.

## Supporting Information

S1 Dataset Lara etal dataset. (SAV)

S1 File Table A. CONSORT 2010 checklist; Table B. TIDieR (Template for Intervention Description and Replication) Checklist; Box A. Description of the Living, Eating, Activity and Planning through retirement (LEAP) intervention; Table C Nutritional intakes before and after interventions.
(DOCX)

S1 Protocol Protocol for LiveWell pilot RCT. (DOCX)
