# Supplementary material for: Pilot Randomised Controlled Trial of a Web-Based Intervention to Promote Healthy Eating, Physical Activity and Meaningful Social Connections Compared with Usual Care Control in People of Retirement Age Recruited from Workplaces
Source: PLoS One. 2016 Jul 29;11(7):e0159703. doi: 10.1371/journal.pone.0159703 (PMC4966951; doi:10.1371/journal.pone.0159703)
Supplement: S1 File — Table A. CONSORT 2010 checklist; Table B. TIDieR (Template for Intervention Description and Replication) Checklist; Box A. Description of the Living, Eating, Activity and Planning through retirement (LEAP) intervention; Table C Nutritional intakes before and after interventions. (DOCX) [file pone.0159703.s002.docx]

**S1 File**

**Table A. CONSORT 2010 checklist of information to include when reporting a randomised trial***

**
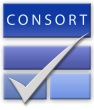
**

| **Section/Topic** | **Item No** | **Checklist item** | **Reported on page No** |
| --- | --- | --- | --- |
| **Title and abstract** | | | |
|  | 1a | Identification as a randomised trial in the title | 1 |
|  | 1b | Structured summary of trial design, methods, results, and conclusions (for specific guidance see CONSORT for abstracts) | 2 |
| **Introduction** | | | |
| Background and objectives | 2a | Scientific background and explanation of rationale | 4-5 |
|  | 2b | Specific objectives or hypotheses | 5 |
| **Methods** | | | |
| Trial design | 3a | Description of trial design (such as parallel, factorial) including allocation ratio | 6 |
|  | 3b | Important changes to methods after trial commencement (such as eligibility criteria), with reasons | N/A |
| Participants | 4a | Eligibility criteria for participants | 8 |
|  | 4b | Settings and locations where the data were collected | 7 |
| Interventions | 5 | The interventions for each group with sufficient details to allow replication, including how and when they were actually administered | refer to TIDieR checklist |
| Outcomes | 6a | Completely defined pre-specified primary and secondary outcome measures, including how and when they were assessed | 10-13 |
|  | 6b | Any changes to trial outcomes after the trial commenced, with reasons | N/A |
| Sample size | 7a | How sample size was determined | N/A |
|  | 7b | When applicable, explanation of any interim analyses and stopping guidelines | N/A |
| Randomisation: |  |  |  |
| Sequence generation | 8a | Method used to generate the random allocation sequence | 6 |
|  | 8b | Type of randomisation; details of any restriction (such as blocking and block size) | 6 |
| Allocation concealment mechanism | 9 | Mechanism used to implement the random allocation sequence (such as sequentially numbered containers), describing any steps taken to conceal the sequence until interventions were assigned | 6 |
| Implementation | 10 | Who generated the random allocation sequence, who enrolled participants, and who assigned participants to interventions | 6 |
| Blinding | 11a | If done, who was blinded after assignment to interventions (for example, participants, care providers, those assessing outcomes) and how | 6 |
|  | 11b | If relevant, description of the similarity of interventions | 8-10 |
| Statistical methods | 12a | Statistical methods used to compare groups for primary and secondary outcomes | 12-13 |
|  | 12b | Methods for additional analyses, such as subgroup analyses and adjusted analyses | 13 |
| **Results** | | | |
| Participant flow (a diagram is strongly recommended) | 13a | For each group, the numbers of participants who were randomly assigned, received intended treatment, and were analysed for the primary outcome | 26-27 |
|  | 13b | For each group, losses and exclusions after randomisation, together with reasons | 14 |
| Recruitment | 14a | Dates defining the periods of recruitment and follow-up | 6 |
|  | 14b | Why the trial ended or was stopped | N/A |
| Baseline data | 15 | A table showing baseline demographic and clinical characteristics for each group | 27 |
| Numbers analysed | 16 | For each group, number of participants (denominator) included in each analysis and whether the analysis was by original assigned groups | 14 |
| Outcomes and estimation | 17a | For each primary and secondary outcome, results for each group, and the estimated effect size and its precision (such as 95% confidence interval) | 15-19 |
|  | 17b | For binary outcomes, presentation of both absolute and relative effect sizes is recommended | N/A |
| Ancillary analyses | 18 | Results of any other analyses performed, including subgroup analyses and adjusted analyses, distinguishing pre-specified from exploratory | 15-19 |
| Harms | 19 | All important harms or unintended effects in each group (for specific guidance see CONSORT for harms) | N/A |
| **Discussion** | | | |
| Limitations | 20 | Trial limitations, addressing sources of potential bias, imprecision, and, if relevant, multiplicity of analyses | 20-21 |
| Generalisability | 21 | Generalisability (external validity, applicability) of the trial findings | 20-21 |
| Interpretation | 22 | Interpretation consistent with results, balancing benefits and harms, and considering other relevant evidence | 21-23 |
| **Other information** | | |  |
| Registration | 23 | Registration number and name of trial registry | 3 |
| Protocol | 24 | Where the full trial protocol can be accessed, if available | 3 |
| Funding | 25 | Sources of funding and other support (such as supply of drugs), role of funders | 24-25 |

*We strongly recommend reading this statement in conjunction with the CONSORT 2010 Explanation and Elaboration for important clarifications on all the items. If relevant, we also recommend reading CONSORT extensions for cluster randomised trials, non-inferiority and equivalence trials, non-pharmacological treatments, herbal interventions, and pragmatic trials. Additional extensions are forthcoming: for those and for up to date references relevant to this checklist, see [www.consort-statement.org](http://www.consort-statement.org).

**
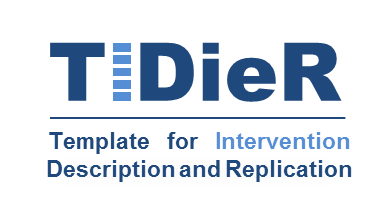
**

**Table B. The TIDieR (Template for Intervention Description and Replication) Checklist*:**

Information to include when describing an intervention and the location of the information

| **Item number** | **Item** | **Where located **** | |
| --- | --- | --- | --- |
|  |  | Primary paper  (page or appendix  number) | Other ^†^ (details) |
|  | **BRIEF NAME** |  |  |
| **1.** | Provide the name or a phrase that describes the intervention. | 8-10 | ______________ |
|  | **WHY** |  |  |
| **2.** | Describe any rationale, theory, or goal of the elements essential to the intervention. | 5, 9 | _____________ |
|  | **WHAT** |  |  |
| **3.** | Materials: Describe any physical or informational materials used in the intervention, including those provided to participants or used in intervention delivery or in training of intervention providers. Provide information on where the materials can be accessed (e.g. online appendix, URL). | 8-10; 42-45 | _____________ |
| **4.** | Procedures: Describe each of the procedures, activities, and/or processes used in the intervention, including any enabling or support activities. | 8-10; 42-45 | _____________ |
|  | **WHO PROVIDED** |  |  |
| **5.** | For each category of intervention provider (e.g. psychologist, nursing assistant), describe their expertise, background and any specific training given. | 8 | _____________ |
|  | **HOW** |  |  |
| **6.** | Describe the modes of delivery (e.g. face-to-face or by some other mechanism, such as internet or telephone) of the intervention and whether it was provided individually or in a group. | 8 | _____________ |
|  | **WHERE** |  |  |
| **7.** | Describe the type(s) of location(s) where the intervention occurred, including any necessary infrastructure or relevant features. | 6 | _____________ |
|  | **WHEN and HOW MUCH** |  |  |
| **8.** | Describe the number of times the intervention was delivered and over what period of time including the number of sessions, their schedule, and their duration, intensity or dose. | 8 | _____________ |
|  | **TAILORING** |  |  |
| **9.** | If the intervention was planned to be personalised, titrated or adapted, then describe what, why, when, and how. | 8-10 | _____________ |
|  | **MODIFICATIONS** |  |  |
| **10.^ǂ^** | If the intervention was modified during the course of the study, describe the changes (what, why, when, and how). | N/A | _____________ |
|  | **HOW WELL** |  |  |
| **11.** | Planned: If intervention adherence or fidelity was assessed, describe how and by whom, and if any strategies were used to maintain or improve fidelity, describe them. | 11 | _____________ |
| **12.^ǂ^** | Actual: If intervention adherence or fidelity was assessed, describe the extent to which the intervention was delivered as planned. | 14-15 | _____________ |

** **Authors** - use N/A if an item is not applicable for the intervention being described. **Reviewers** – use ‘?’ if information about the element is not reported/not sufficiently reported.

† If the information is not provided in the primary paper, give details of where this information is available. This may include locations such as a published protocol or other published papers (provide citation details) or a website (provide the URL).

ǂ If completing the TIDieR checklist for a protocol, these items are not relevant to the protocol and cannot be described until the study is complete.

* We strongly recommend using this checklist in conjunction with the TIDieR guide (see *BMJ* 2014;348:g1687) which contains an explanation and elaboration for each item.

* The focus of TIDieR is on reporting details of the intervention elements (and where relevant, comparison elements) of a study. Other elements and methodological features of studies are covered by other reporting statements and checklists and have not been duplicated as part of the TIDieR checklist. When a **randomised trial** is being reported, the TIDieR checklist should be used in conjunction with the CONSORT statement (see [www.consort-statement.org](http://www.consort-statement.org)) as an extension of **Item 5 of the CONSORT 2010 Statement.** When a **clinical trial** **protocol** is being reported, the TIDieR checklist should be used in conjunction with the SPIRIT statement as an extension of **Item 11 of the SPIRIT 2013 Statement** (see [www.spirit-statement.org](http://www.spirit-statement.org)). For alternate study designs, TIDieR can be used in conjunction with the appropriate checklist for that study design (see [www.equator-network.org](http://www.equator-network.org)).

**Box A. Description of the Living, Eating, Activity and Planning through retirement (LEAP) intervention**

*LEAP intervention*. Participants in the LEAP intervention arm were instructed, via email, to access the website and register. They were also mailed a dual-axis pedometer (Omron HJ203) to be used during intervention. LEAP is a web-based intervention using a responsive design so that it can be accessed on a PC, tablet or mobile phone. The intervention comprised five modules of tools, activities and resources for the intervention user. There were also sections for user registration, LEAP overview, and user diary and user dashboard. The intervention content presented was personalised to the user on the basis of the information they provide at different stages. LEAP makes suggestions about which modules may be most beneficial for the user based on the information about the user’s individual needs and situation. However, the user determined the route to take through the intervention, choosing the order and the modules they prefer, and skipping or revisiting modules as desired. Thus, each user’s experience of the intervention is tailored to their preferences.

LEAP includes five modules: 1) Time, 2) Changing Work, 3) Moving More, 4) Being Social, 5) Eating Well, as well a diary and a dashboard sections. In the ‘Time’ module the user reflected on how they spend their time currently and how they would like to at a later stage in their retirement transition. Users were asked to think specifically about time spent working, caring for others, pursuing hobbies, being physically and socially active, doing household duties and having undefined ‘free time’.

Although reflection on the use of time was not initially a target for the LiveWell programme, analysis of the qualitative data indicated that assistance with reflecting on current and future time use was important for people within the retirement transition, especially with regard to thinking through the possibilities for various lifestyle behaviours, goals and aspirations. The format of this module was developed further through the co-design workshops to include a user-friendly graphical display of time use.

The ‘Changing Work’ module allowed the user to consider their financial and work situation as they move through the retirement transition. Users who were not fully retired (working full or part time) were able to explore whether they could afford to retire when they would like to or whether reducing their working hours would be a possible solution. Already retired users were able to consider their income and expenses and whether they would like to look for part-time work or start their own business. The module uses a range of tools including calculating cost of living, likely income and expenditure, and displaying the results in graphical formats. The tool prompts the user to consider the contribution of their state pension and bus pass and states when these are applicable given current legislation and the user’s date of birth and gender. There were also useful links to external resources for the user to access for a more detailed personal assessment. Like the ‘time’ module , work transitions were not originally a target for intervention development in LiveWell. However analysis of our qualitative data indicated that finances and modes of work transition shaped how people experienced retirement, and set the conditions for lifestyle behaviours.

The ‘Moving More’ module supported the user to move more and sit less. Users were provided with a dual-axis pedometer (Omron HJ203) at the beginning of the intervention to measure their step count. The module encouraged the user to self-monitor their daily step count and to set daily step goals. The user was prompted to set outcome goals for being more active (i.e., lose weight, feel happier etc.) and to explore different ways of being more active. The user could schedule when they will be more active in a diary, explicitly stating when, where and with whom they do a particular activity (i.e., action planning). Finally the user was presented with potential barriers to doing the activities they have declared that they wished to undertake and encouraged to identify potential solutions to the problems. The user was reminded to regularly return to this module to update their daily step count, review their goals, schedule new activities and reconsider the barriers and solutions to being more active. The user could share what they have done in this module via email, Facebook or twitter, and could print a summary to serve as a reminder for themselves.

The ‘Being Social’ module explored the potential benefits of having a meaningful occupation or role and spending time with friends, family and work colleagues. In the social roles tool the user was asked if they would like to consider paid, unpaid or both forms of work and is provided with some case studies to help them consider their preferences. If the user chose paid work they were reminded to explore the ‘work transitions’ module but are also given links to relevant external sites (e.g. business link). If the user selected non-paid work they completed a brief questionnaire designed to stimulate reflection on the type of role that the user might pursue and to elicit information regarding occupation or role preferences. The user was prompted to print their list of role preferences and is then presented with links to relevant external organisations that offer volunteering placements. In the social relationships tool, the user was prompted to explore the relationships that are important to them which are mapped out visually. The user was then prompted to think how these relationships might change through the retirement transition, and to add any additional relationship ‘types’ (e.g. to make new friends) that they might want to develop in the future. The tool helped the user to identify opportunities to develop these relationships through several tools. In the first instance the user was invited to enter individual names within each relationship category to help personalise later steps in the module. The tool then presented a list of potential social activities for each relationship type, which could be filtered based on preferences of cost, accessibility (e.g. for young children or people with limited mobility), and intensity (level of physical activity involved). The activities presented by the tool were those that suit the particular relationship type currently selected by the user (e.g. grandchildren). The user could save activities for later consideration, or add activities (with specific individuals or as relationship type) to their diary explicitly stating when, where and with whom they do a particular activity (i.e., action planning).

The ‘Eating Well’ module encouraged the user to consider their current diet and to explore ways in which it might be changed to correspond more closely to a Mediterranean-style eating pattern[[30](#_ENREF_30)]. Initially, users were invited to respond to a 14-item validated questionnaire designed to capture key facets of their current diet in relation to the principles of Mediterranean eating [[31](#_ENREF_31)]. Then they received brief feedback specifically tailored to the questionnaire items on which they did not score optimally. Users had the chance to prioritise and refine the feedback further before receiving ideas for meals (snacks, drinks, breakfast, light meals and main meals) that put the feedback into practice. Users could filter the meal ideas they viewed by dietary preference, cost, cooking skills and time. They had the opportunity to save meal ideas in a virtual ‘recipe book’ if they would like to try them, and they were encouraged to add meal ideas to their diary, stating when, where and with whom they would eat. Users were also prompted to reflect on the goals they would like to achieve by eating better; they were further prompted to consider barriers that may obstruct their efforts to put into practice the feedback they have received. Upon exiting the module, they received a reminder of the feedback on their current diet and the dietary change goals that they had identified.

The ‘diary’ section presented the activities that the user had scheduled for the next two weeks. This information could be downloaded to the user’s computer calendar or printed.

The ‘dashboard’ section presented a summary of the activities, tools and resources the user had engaged with or has saved to view later. It prompted the user to revisit modules to report on the activities they have engaged with, revise their goals, schedule new activities etc.

**Table C Nutritional intakes before and after interventions**

|  | **Control (n=22)** | | **Intervention (LEAP) (n=48)** | |
| --- | --- | --- | --- | --- |
|  | **Before**  Mean (95% CI) | **After**  Mean (95% CI) | **Before**  Mean (95% CI) | **After**  Mean (95% CI) |
| Energy intake MJ/d | 8.9 (7.7 to 10.2) | 8.9 (7.9 to 9.9) | 8.9 (8.0 to 9.8) | 8.5 (7.8 to 9.2) |
| Fat (g/day) | 86.9 (73.5 to 100.2) | 88.1 (74.9 to 101.4) | 82.4 (72.9 to 91.8) | 79.6 (70.4 to 88.8) |
| CHO (g/day) | 234.1 (194.2 to 273.9) | 231.9 (200.0 to 263.9) | 248.1 (219.9 to 276.3) | 239.9 (217.9 to 262.1) |
| Protein (g/day) | 80.0 (69.8 to 90.2) | 83.1 (72.4 to 93.8) | 80.4 (73.2 to 87.6) | 81.0 (73.7 to 88.4) |
| Alcohol (g/day) | 15.2 (7.3 to 23.1) | 11.2 (4.6 to 17.8) | 17.2 (11.6 to 22.8) | 13.4 (8.9 to 17.9) |
