# Supplementary material for: Pilot Randomised Controlled Trial of a Web-Based Intervention to Promote Healthy Eating, Physical Activity and Meaningful Social Connections Compared with Usual Care Control in People of Retirement Age Recruited from Workplaces
Source: PLoS One. 2016 Jul 29;11(7):e0159703. doi: 10.1371/journal.pone.0159703 (PMC4966951; doi:10.1371/journal.pone.0159703)
Supplement: S1 Protocol — (DOCX) [file pone.0159703.s003.docx]

**DEVELOPING INTERVENTIONS TO ENHANCE HEALTH AND WELLBEING IN LATER LIFE: PROTOCOL FOR THE LIVEWELL PROGRAMME PILOT RANDOMISED CONTROLLED TRIAL**

# STUDY BACKGROUND

In contrast with the wealth of observational data supporting the hypothesis that health and

wellbeing in later life are influenced strongly by behavioural factors and social conditions, there is a dearth of evidence about interventions that are effective in promoting improved health and wellbeing in later life. Prerequisites for the development of such interventions include identification of: i) intervention modalities that are expected to be effective, culturally appropriate and cost effective; ii) windows of opportunity when interventions are likely to be most effective; and iii) outcome measures that are responsive to the interventions and can measure change in health and wellbeing. Life stage transitions involve changes in lifestyle and thus present key opportunities for behaviour change interventions; the LiveWell research programme aims to develop evidence-based, acceptable and scalable interventions to improve health and wellbeing in the retirement transition.

This programme of research follows the MRC framework for the development and evaluation of complex interventions (Craig et al, 2010). First, we systematically reviewed the effectiveness of interventions to change key health behaviours and social factors of people in the retirement transition. We have found that interventions with people of retirement age can effectively promote components of the Mediterranean diet (Lara et al, unpublished), physical activity (Hobbs et al, 2013) and explicit social roles (Heaven et al, 2013). We have also identified the intervention modalities and intervention content (behaviour change techniques; Michie et al., 2011) associated with intervention effects. We have also conducted qualitative research with people in the retirement transition to explore how wellbeing is defined and how best to intervene to promote health and wellbeing. Second, using co-design methods (Bate & Robert, 2007; NHS Institute for Innovation and Improvement, 2009) with intervention stakeholders (older adults as potential intervention users, intervention providers or commissioners), we integrated the evidence from the systematic reviews and qualitative research to develop and prototype new intervention ideas. In collaboration with a digital marketing and design company (Hippo Creative and Cloud Data Service), a web-based intervention platform has been developed using an iterative design and development process. Potential intervention users have been involved at all stages using co-design methods, providing information on intervention feasibility, acceptability and usability. The final intervention is called LEAP “living, eating, activity and planning through retirement”, and will now be formally tested for feasibility and acceptability in a pilot randomised controlled trial (RCT). This trial will be registered with International Standard Randomised Controlled Trial Number Register (ISRCTN).

## The LEAP intervention

LEAP is a web-based intervention using a responsive design so that it can be accessed on a PC, tablet or mobile phone. The intervention comprises five modules of tools, activities and resources for the intervention user. There are also sections for user registration, LEAP overview, user diary and user dashboard. The intervention content that is presented is personalised to the user on the basis of the information they provide at different stages. The user determines the route they take through the intervention, choosing which modules to complete in what order, and skipping or revisiting modules as desired. However, the intervention uses information about the user’s individual needs and situation to make suggestions about which modules may be most beneficial for the user. Thus, each user’s experience of the intervention is tailored to their preferences.

### Virtual Mentor

As part of the co-design phase, we explored what an intervention for people in the retirement transition could look like and how it could be delivered. A priority for the research team was to develop a digital-based intervention which may be more compatible with modes of accessing information in future cohorts of older people (e.g. by smart phone) rather than traditional paper and pencil methods. A digital intervention also has greater potential for wide-scale use in the target population. A web-based intervention was seen to be an acceptable mode of delivery by older adults. Some older adults emphasised that a web-based intervention would allow them to access the intervention in their own homes and at a time convenient to them. However, the potential risks associated with a web-based intervention were also expressed; some older adults felt that they might not engage with a website and feel unsupported. Older adults in the co-design workshops identified that having access to a mentor could help to explore retirement transition options and lifestyle behaviours. In order to produce a cost-effective, digital-based intervention, the research team developed a range of ‘virtual mentors’ to guide intervention users through the website. The idea of a virtual mentor was developed further and tested by older adults. The mentoring feature was found to be useful.

When the user registers with LEAP they are invited to choose a mentor to guide and support them through the intervention. There are eight animated mentors to choose from; four males and four females, each of whom is presented alongside their brief personal background. The mentor ‘speaks’ to the user as they move through the intervention, explaining the purpose of each module and activity, providing instructions and directing them to resources. A user can choose to mute the mentor’s voice and just read instructions if they prefer.

### LEAP modules and features

1. The ‘registration’ section is applicable when the user first accesses LEAP. This module includes a sign up page and a short registration questionnaire about retirement status (fully retired, semi-retired or not yet retired), and feelings about physical activity, dietary behaviours, and social relationships.
2. The ‘LEAP overview’ section provides the user with an overview of the objectives of each module and guidance on the general functions and features of LEAP, including the ability to choose and subsequently amend personal preferences.
3. The ‘Time’ module encourages the user to reflect on how they spend their time currently and how they would like to at a later stage in their retirement transition. They are asked to think specifically about time spent working, caring for others, pursuing hobbies, being physically and socially active, doing household duties and having undefined ‘free time’. Although reflection on the use of time was not initially a target for the LiveWell programme, analysis of the qualitative data indicated that assistance with reflecting on current and future time use was important for people within the retirement transition, especially with regard to thinking through the possibilities for various lifestyle behaviours, goals and aspirations. The format of this module was developed further through the co-design workshops to include a user-friendly graphical display of time use.
4. The ‘Changing Work’ module allows the user to consider their financial and work situation as they move through the retirement transition. Users who are not fully retired (working full or part time) can explore whether they can afford to retire when they would like to or whether reducing their working hours would be a possible solution. Users who have already retired may consider their income and expenses and whether they would like to look for part-time work or start their own business. The module uses a range of tools including calculating cost of living, likely income and expenditure, and displaying the results in graphical formats. The tool prompts the user to consider the contribution of their state pension and bus pass and states when these are applicable given current legislation and the user’s date of birth and gender. There are also useful links to external resources for the user to access for a more detailed personal assessment. Like the ‘time’ module – work transitions were not originally a target for intervention in LiveWell. However analysis of our qualitative data indicated that finances and modes of work transition shaped how people experienced retirement, and set the conditions for lifestyle behaviours.
5. The ‘Moving More’ module supports the user to move more and sit less. Users are provided with a pedometer at the beginning of the intervention to measure their step count. The module encourages the user to self-monitor their daily step count and to set daily step goals. The user is prompted to set outcome goals for being more active (i.e., lose weight, feel happier etc.) and to explore different ways of being more active. The user can schedule when they will be more active in a diary, explicitly stating when, where and with whom they do a particular activity (i.e., action planning). Finally the user is presented with potential barriers to doing the activities they have said they would like to and encouraged to identify potential solutions to the problems. The user is reminded to regularly return to this module to update their daily step count, review their goals, schedule new activities and reconsider the barriers and solutions to being more active. The user can share what they have done in this module via email, Facebook or twitter, and can print a summary to serve as a reminder for themselves.
6. The ‘Being Social’ module explores the potential benefits of having a meaningful occupation or role and spending time with friends, family and work colleagues. In the social roles tool the user is asked if they would like to consider paid, unpaid or both forms of work and is provided with some case studies to help them consider their preferences. If the user chooses paid work they are reminded to explore the ‘work transitions’ module but are also given links to relevant external sites (e.g. business link). If the user selects non-paid work they complete a brief questionnaire designed to stimulate reflection on the type of role that the user might pursue and to elicit information regarding occupation or role preferences. The user is prompted to print their list of role preferences and is then presented with links to relevant external organisations that offer volunteering placements. In the social relationships tool, the user is prompted to explore the relationships that are important to them which are mapped out visually. The user is then prompted to think how these relationships might change through the retirement transition, and to add any additional relationship ‘types’ (e.g. to make new friends) that they might want to develop in the future. The tool helps the user to identify opportunities to develop these relationships through several tools. In the first instance the user is invited to enter individual names within each relationship category to help personalise later steps in the module. The tool then presents a list of potential social activities for each relationship type, which can be filtered based on preferences of cost, accessibility (e.g. for young children or people with limited mobility), and intensity (level of physical activity involved). The activities presented by the tool are those that suit the particular relationship type currently selected by the user (e.g. grandchildren). The user can save activities for later consideration, or add activities (with specific individuals or as relationship type) to their diary explicitly stating when, where and with whom they do a particular activity (i.e., action planning).
7. The ‘Eating Well’ module encourages the user to consider their current diet and explore ways in which it might be changed to correspond more closely to a Mediterranean-style eating pattern. Initially, users are invited to respond to a 14-item validated questionnaire designed to capture key facets of their current diet in relation to the principles of Mediterranean eating (Martinez-Gonzalez et al, 2012). They then receive brief feedback specifically tailored to the questionnaire items on which they did not score optimally. Users have the chance to prioritise and refine the feedback further before receiving ideas for meals (snacks, drinks, breakfast, light meals and main meals) that put the feedback into practice. Users can filter the meal ideas they view by dietary preference, cost, cooking skills and time. They have the opportunity to save meal ideas in a virtual ‘recipe book’ if they would like to try them, and they are encouraged to add meal ideas to their diary, stating when, where and with whom they will eat. Users are also prompted to reflect on the goals they would like to achieve by eating better; they are further prompted to consider barriers that may obstruct their efforts to put into practice the feedback they have received. Upon exiting the module, they receive a reminder of the feedback they received and the outcome goals they identified.
8. The ‘diary’ section presents the activities that the user has scheduled for the current week and the following week. This information can be downloaded to the user’s computer calendar or printed.
9. The ‘dashboard’ section presents a summary of the activities, tools and resources the user has engaged with or has saved to view later. It prompts the user to revisit modules to report on the activities they have engaged with, revise their goals, schedule new activities etc.

### Notifications

In the ‘LEAP overview’ section, users will be asked to opt-in to receive via email a weekly bulletin summarising the information that is displayed on their dashboard. This bulletin will act as a prompt for the user to revisit LEAP and engage further with the modules. Users can amend whether they receive this bulletin or not at any time by modifying their personal preferences.

## Healthy Ageing Phenotype

The lack of outcome measures which capture the essential components of the healthy ageing phenotype (HAP), as distinct from measuring disability or ill-health, is a significant impediment to the evaluation of interventions in this area. In this programme of research we have defined a suite of outcome measures, including relevant biomarkers, and identified tools appropriate for capturing the HAP (Lara et al, 2013). We have selected a subset of these measures and tools that may be appropriate to use in a pragmatic RCT with long-term follow up. The feasibility and acceptability of these measures has yet to be determined and therefore will be formally assessed in this pilot RCT alongside more proximal outcomes of the intervention modules (i.e. diet, physical activity and social roles).

# SCIENTIFIC RATIONALE and research aims

Before commencing an appropriately powered RCT of the LEAP intervention including an assessment of the HAP, information is needed about the number of eligible participants and the rates of recruitment, retention and data completion. This information will help establish if LEAP will be used by older adults and whether the HAP outcome measures are fit for purpose.

## **Research Aims**

To provide early markers of how the intervention is used, data on physical activity, dietary and social factors, and variability of response to LEAP in order to inform effect and sample size estimates. Qualitative methods will capture additional information including reasons for participation, willingness of participants to be randomised to the intervention, acceptability of data collection and reported use and acceptability of the intervention. The pilot trial will also collect information on the acceptability, feasibility and compliance with the HAP battery assessment procedures.

# STUDY DESIGN

This study is a pilot, individual randomised, single blinded, controlled trial of a web-based lifestyle intervention for people in the retirement transition versus a minimal intervention comparator. Embedded in the trial design is a qualitative process evaluation and an assessment of feasibility and acceptability of a battery of HAP outcome measures.

A total of **90** participants will be recruited and participants will be randomly allocated to one of two conditions:

1. LEAP intervention
2. Minimal intervention control comparator

An allocation ratio of 2:1 will be used for the intervention group compared to the control comparator group.

## LEAP intervention

A total of **60** participants will be randomised to receive the LEAP intervention. Thirty participants are usually sufficient for feasibility testing of an intervention (Lancaster et al, 2004); however, LEAP is a personalised intervention with diverse modules, tools and activities that are tailored to the needs and desires of the user at their individual stage in the retirement transition. The number of potential routes through LEAP and the heterogeneity of the target population are therefore substantial, and thus we will allocate more participants to receive LEAP compared to the control comparator.

## Control Comparator

A total of **30** participants will be randomly allocated to the control comparator which will involve them being emailed a direct link to the NHS choices ‘LiveWell’ website (http://www.nhs.uk/LiveWell/Pages/Livewellhub.aspx). The email will encourage the participants to access the health resources and information on the pages labelled *men’s health 40-60, men’s health 60-plus, women’s health 40-60, women’s health 60-*plus, as appropriate.

## Randomisation

Method of randomisation

Eligible participants will be randomly assigned (in ratio of 2:1) to either LEAP or Control. Randomisation will be undertaking with randomly chose randomisation blocks (3, 6, 9) to maintain balance. All personnel and participants will be blinded to treatment allocation; only a member of the team not involved in aspects of assessment or interventions will have access to treatment allocation. Length of follow-up

This is a pilot feasibility trial and one of its objectives is to collect data about the number of eligible participants and the rates of recruitment, retention and data completion. Study attrition is largely seen within the first month of a trial, therefore we will have a two month follow-up from the start of the intervention, which will give a good indicator of likely attrition in a future definitive RCT with a longer follow-up. We will also collect data on dietary patterns, physical activity and social relationships to identify variability in response to the intervention. The trial is not powered to detect change in these variables in response to the intervention, thus, adopting a longer follow-up period of six or 12 months in order to examine behavioural maintenance is not required. The behavioural data will however be used to inform effect and sample size estimates for a future definitive trial. Figure 1 illustrates participant flow through the trial in accordance with the CONSORT guidelines.


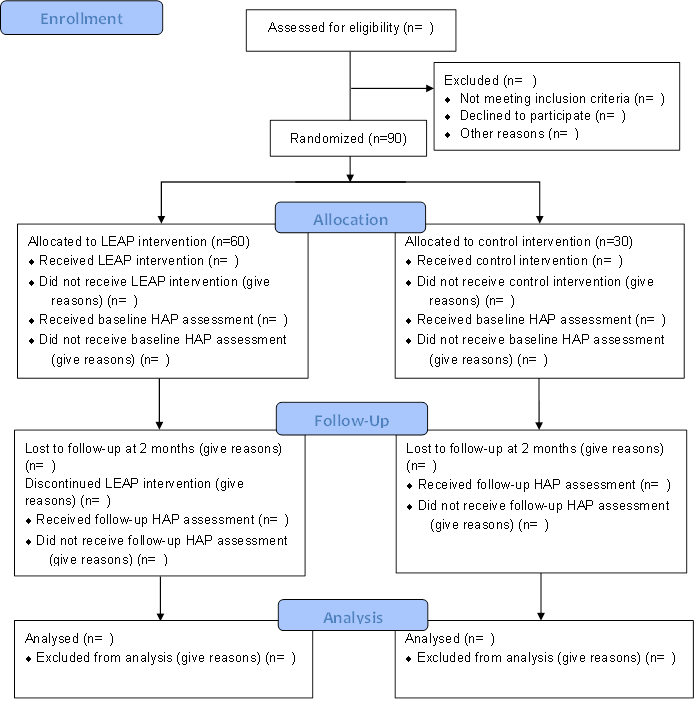


**Fig 1. CONSORT 2010 RCT flow diagram**

# Research questions

This pilot trial addresses the following specific research questions:

## Recruitment and retention

1. What is the trial recruitment rate? (i.e., what percentage of people eligible to take part, in the specified geographical location, accepts the invitation to participate)?
2. What percentage of participants are recently retired compared to the percentage that are not yet retired?
3. Does the recruitment strategy result in the successful inclusion of participants from blue collar, manual or physical labour occupational backgrounds?
4. If the recruitment strategy via the community is adopted, in addition to the strategy via employers (see Section 5), then do the recruitment rate and the characteristics of the recruited sample differ for each strategy?
5. What is the trial retention rate? (i.e., what percentage of participants from each intervention arm remains in the trial at each follow-up assessment point?)

## Compliance with intervention procedures and use of the intervention

1. Which LEAP pages/modules/tools are used, how many times, for how long in total and on each occasion, and in what order?
2. To what extent do participants complete the activities and use all the tools within each module?
3. Is usage a personalised experience or are there patterns in usage across the sample?
4. What platform is used to access LEAP (PC, tablet and/or smartphone) and is the use of multiple platforms moderated by sociodemographic factors?
5. What percentage of participants opts to receive email notifications in the form of a weekly bulletin?
6. What percentage of participants responds to notifications? (i.e., logs in and updates personal information, recent behaviour, reviews goals etc.)
7. Which participants wear the pedometer for the duration of the trial and what is their pattern of usage?

## Compliance with intervention measurement procedures

1. What percentage of participants use the *Axivity* device according to protocol at baseline and post-intervention assessment?
2. What percentage of *Axivity* data is missing/incomplete or unreliable?
3. How many devices are lost at baseline and a follow-up assessment?
4. What percentage of participants completes the *24 hour recall diet* assessment at baseline and post-intervention assessment?
5. What percentage of the *24 hour recall* data is missing/incomplete or unreliable?
6. What percentage of participants completes the online *social* measures at baseline and post-intervention assessment?
7. What percentage of the online *social* measure data is missing/incomplete or unreliable?

## Validity of intervention measures and responsiveness to detect change

1. Is there preliminary data that suggests that the Axivity device is sensitive to detect change in all ambulatory behaviour in response to the intervention in this population?
2. Is there preliminary data that suggests that the 24 hour dietary recall measure is sensitive to detect change in response to the intervention in this population?
3. Is there preliminary data that suggests that the social measure is sensitive to detect change in response to the intervention in this population?

## Compliance with HAP battery measurement procedures

1. What is the mean (SD) length of time required to complete the full HAP assessment?
2. What percentage of participants completes the full HAP outcome assessment at each time point and how long does it take?
3. What percentage of outcome data is missing?

# Participant Recruitment

Primarily, individuals will be recruited through employers. We will work with Human Resource departments of medium to large businesses in the region (e.g. Nissan, Post Office, Local Authority, Department for Work and Pensions) and with Health Improvement Specialists (Scott Lloyd and Joanne Benson), who have established relationships with local employers. We will aim to recruit individuals in full or part time work, and individuals who have recently retired (within the last 2 years). We cannot predict how easily we will access recently retired individuals via employers, therefore one of the aims of this pilot is to test this aspect of recruitment. If recruiting recently retired individuals is problematic, then we will adopt an additional recruitment strategy in the community. We will work with older people’s organisations (e.g. AgeUK, Gateshead Older People’s Forum and University of the Third Age) and advertise within local communities (e.g. supermarkets, Citizen Advice Bureau offices, libraries and community centre notice boards), with the aim to recruit individuals who have retired but not joined any national organisations, community or advocacy groups. Although we cannot predict who may be recruited via this strategy in the community, it increases the opportunity to include individuals in the trial who have experienced greater levels of disadvantage (e.g. through low incomes and poorer social networks) and who might, therefore, represent a key group of potential service users. Recruitment will begin in February and continue until April (3 months) unless recruitment targets (n=90) are met within a shorter timescale.

## Inclusion Criteria

A male or female who has retired in the last 2 years, is planning to retire in the next 2 years or who has reduced working hours in anticipation for retirement. The individual must own a PC, tablet or smartphone, and have reliable internet access at home, work or mobile 3G.

## Exclusion Criteria

Participants who cannot comprehend and speak fluent English will be excluded from participation. Similarly, participants will be excluded if they score ≥20 on the Center for Epidemiologic Studies Depression Scale (CESD), a measure of recent depressed feelings and behaviours, and/or scoring two or more standard deviations below the mean for age, gender, and education on the Paired Associate Learning (PAL), a measure of memory ability. Participants who are concerned about participating due to a health condition will be advised to consult their GP about their fitness and ability to participate: this also encompasses any participants with a probable severe mental health condition (as assessed by a brief telephone screening questionnaire). Volunteers with very high blood pressure (i.e. >180mmHg/>110) identified during the first heath assessment session will be advised to consult their GP as soon as possible; and discuss the potential benefit of taking part in this study. Volunteers who are concerned about participating due to a health condition will be advised to consult their GP about their fitness and ability to participate, and those advised not to participate will be excluded. Sampling

We will attempt to include participants who vary on the following criteria: retirement status; chronological age (younger vs older); gender; work category (manual, semiskilled or technical, managerial or professional), and work-related activity (physically demanding or sedentary). We will weight recruitment in favour of blue collar, manual and lower managerial occupations. To facilitate recruitment across these categories we will attempt to recruit employers who provide different forms of employment within the business (e.g. both office and blue collar positions), and if necessary, recruit employers that employ people in particular occupations if these are under-represented in our sample (e.g. high in work-related physical activity).

# Outcome Assessment

## Assessment Times

- Baseline before randomisation
- 2 months after starting the intervention.

## Baseline and 2-month Follow-up Assessments

The assessments at baseline and at 2-months are identical. The assessments will include behavioural measures of physical activity and inactivity, dietary intake and social relationships, and HAP measures. It is estimated that these measurements can be completed within 2 hours. The assessments will take place at the participant’s place of work. Participants who have retired will be asked to attend assessments at their previous place of work, at a local privately hired venue (e.g. community hall), or in their own homes. Assessments will be carried out by a member of the study team trained to administer the various measures. The psychological and social wellbeing questionnaires will be self-reported by the participant. In Appendix A there is the measurement battery protocol, including the list of measures, instructions for their administration and analysis of the data acquired).

# STUDY Procedure

This pilot trial will work through the following steps:

1. The trial administrator and (where appropriate) collaborating Health Improvement Specialists will contact Human Resources departments of local businesses to ask them if they would be willing to advertise the study to their employees or constituents (email, letters, posters in workplace etc) and where appropriate, contact individual employees or members of the organisation directly (e.g. personalised e-mail). If recruitment is also conducted in the community (e.g. in order to recruit people who have retired) then we will also contact community organisations of older adults and display posters in locations that may be frequented by people living in low socio economic neighbourhoods (more likely to have retired from blue collar or manual occupations rather than managerial or professional roles), and which aren’t linked specifically to existing membership or engagement with routine physical activity (e.g. sports centres). Our strategy therefore will be to display posters in low SES wards in Newcastle (e.g. Benwell, Elswick ) in the following locations: pubs; working men’s clubs; libraries; Citizen Advice Bureaus; supermarkets; noticeboards in religious buildings (e.g. churches, mosques) and shop windows.
2. Potential participants who are interested in participating will be asked to make contact with the trial administrator or to agree for the Human Resources department or community organisation to pass on their details to the research team who will then contact the potential participant directly.
3. The trial administrator will contact the Human Resources departments and community organisations in one week if there has not been any contact.
4. Over the telephone, a member of the research team will assess the potential participants for eligibility using the defined inclusion and exclusion criteria.
5. Eligible participants will be emailed or sent a study invitation letter, a participant information sheet and a consent form. They will be informed that one of the study team will make contact with them in the next five working days to discuss their participation in the study and to answer any questions.
6. In the next five days, the trial administrator will telephone each eligible participant to answer any questions and to ascertain whether they would like to accept the invitation to participate. If there are any questions that can’t be answered over the phone by the administrator, a date for follow-up correspondence (either by e-mail or phone according to the preference of the potential participant) will be made. The administrator will request an answer to the question from the relevant trial team personnel, and will then relay this information to the participant during the arranged follow-up correspondence. On verbal consent, the trial administrator will ask that the participant sign the consent form and keep it until one of the research team visits them. The administrator will then schedule an appointment at a convenient time when one of the study team will visit the participant at their home or place of work to obtain the signed informed consent form and conduct the baseline assessment. The researcher will attend the appointment with spare consent forms in case the participant mislaid their original consent form.
7. Baseline assessment visit - after receiving informed consent, a member of the study team will administer the HAP battery assessment as per protocol (Appendix A). The activity monitor device will be attached to the participant, and it will be explained that the device is to be worn continuously for the next 7 days. The participant will be provided with a prepaid stamped address envelope to return the device after 7 days. The 24 hour recall dietary assessment tool procedure will be explained and participant will complete the first of three days to be recorded, and a web link to the social relationships tools will be provided. During this assessment session participants will be asked to provide information on smoking, alcohol consumption and demographic (Attachment 4 – Baseline data form).
8. The trial administrator will be informed by the member of the study team when baseline assessment is complete and the administrator will randomise the participant to one of the trial conditions recording key information (e.g. name, D.O.B. and assigned identification number) into a central database. A pedometer and instructions for its use will be posted to participants allocated to receive the LEAP intervention. The trial administrator will telephone or email the participant a few days later to check that the pedometer was received.
9. Seven days after the trial administrator has confirmed that participants allocated to the LEAP intervention have received the pedometer, the trial administrator will email them a web link to access the intervention. They will also be provided with contact details of members of the study team to troubleshoot technical problems.
10. Immediately after the baseline assessment, the trial administrator will email a web link to the NHS LiveWell resource to participants allocated to receive the control intervention.
11. All participants will be reminded that they will be contacted again in approximately 6 weeks to arrange a follow-up visit by a member of the study team in two months’ time.
12. In the next three days, the trial administrator will telephone or email the participant to check that they received the web link to the intervention. During the call the administrator will arrange a date and time for a qualitative interview with those participants who have been identified as suitable using the purposive sampling procedure described in the qualitative protocol (see section 9). For participants randomised to the intervention group, the interview will be scheduled to take place no sooner than a week following the call to allow the participant to use the LEAP intervention and to ensure the interview is not conflated with use of LEAP. Approximately (n=6-8) appointments will be made with participants in the LEAP arm, and up to 4 appointments with participants in the control, with a further 6-8 and 4 appointments respectively being made toward the end of the trial. This will allow the research team to compare accounts of participation at the start and end of the trial respectively, potentially identifying unexpected participant expectations or disappointments/misunderstandings throughout the trial.
13. Six weeks after receiving the link to the LEAP or control intervention, the trial administrator will telephone participants to schedule an appointment at a convenient time in the next two weeks when one of the study team will visit the participant at their home or place of work to conduct the follow-up assessment. During the call the administrator will arrange a date and time for a qualitative interview with participants (n=6-8 in LEAP; n= 4 in control) who have been identified as suitable for a qualitative interview.
14. Two month follow-up assessment visit – the same procedure and protocols will be followed as were used for the baseline assessment. The members of the study team conducting the follow-up assessments will be blind to the trial condition of each participant.

The administrative steps for the pilot trial are represented in Figure 2.


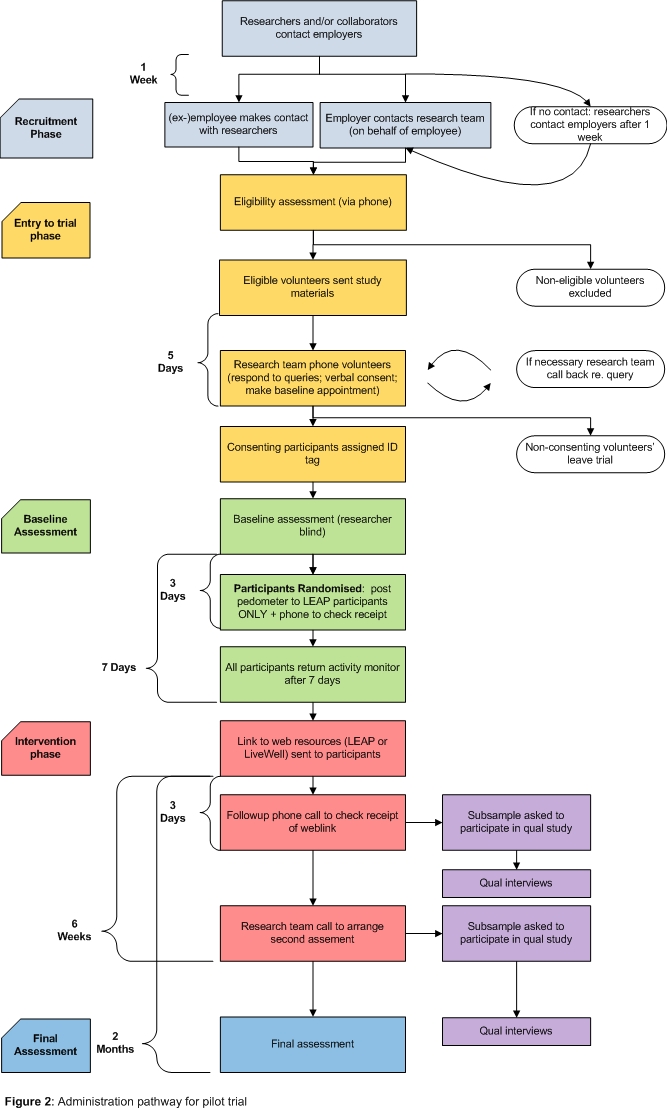


# Data Analysis

*Intervention patterns of use – website analytics*

Data on intervention use and patterns of use will be collected by Cloud Data Service, the company that built the LEAP intervention, using website analytics systems such as Google Analytics. Usage metrics will be compared directly and over time to looks for patterns and trends in the data.

*Statistical analysis of data collected*

Statistical advice and support will be provided by Professor John Mathews’ group from the School of Mathematics and Statistics, Newcastle University.

# Qualitative Assessment

*Objectives*: To conduct qualitative interviews with a purposive sample of participants invited to participate in the pilot trial, to explore reasons for participation or non-participation, experiences of using the intervention, and acceptability of the intervention and assessments

These data will help evaluate the feasibility of a future definitive RCT and explore: opportunities to maximise recruitment and retention; acceptability of data collection including use of the HAP assessment; use of the LEAP intervention including problems of accessibility and function when used within the home or work environment; and data on the acceptability and reported use of each module (diet, PA, social, changing work and time).

## Research Questions addressed by the Qualitative Interviews

With regard to qualitative outcomes in the pilot study, there are three overlapping domains of enquiry: 1) questions relating to participation in the pilot study; 2) questions relating to use of the intervention website; and 3) questions relating to the use of the HAP subset of measurement tools:

### Domain 1: Participation in pilot

1. How did those participating in the pilot come to be recruited? (what did they think about the recruitment strategy, materials etc.)
2. What are the reasons for refusing to participate? (small sub-sample)
3. Why do people agree to take part in the study?
4. What do participants expect from the study? (Are their expectations met?)
5. What are the reasons for trial retention?
6. What are the reasons for trial attrition? (small sub-sample)
7. How do participants feel about being randomised to their arm of the trial (compare and contrast the different conditions)?
8. What are participants’ experiences of the diet, PA and social measures assessment at each time point? (e.g. acceptable? time needed to complete? ease of completion? etc)

Our research questions point to recruitment from 4 groups: 2 arms of the trial, participants who decline to take part in the pilot (e.g. decline to be randomised to a trial arm) but consent to the qualitative component, and participants who drop out of the pilot before the final assessment but consent to a qualitative follow up interview).

### Domain 2: Acceptability and use of intervention tools

1. What do participants expect from the intervention website? (Are their expectations met?)
2. What are the reasons for using some modules and tools but not others?
3. What is useful about each module and tool, and what is not useful? (what could be improved?)
4. How do participants’ experience receiving and responding to notifications and prompts?
5. How do participants’ experience the mentor component of the website?
6. How do participants’ experience the website overall? (i.e., engaging, fun, boring, easy to use, intuitive, innovative etc.?)
7. What do participants anticipate will happen after the pilot study (regarding their behaviours, lifestyle and retirement trajectory?)

### Domain 3: Acceptability and use of HAP battery

1. How do participants’ experience the HAP assessment overall (e.g. time taken, impression of question type, format and delivery)?
2. What components/instruments of the HAP measures were acceptable, and which were difficult to complete (e.g. difficult to understand, repetitive, or ‘simplistic’)?
3. What (if anything) is missing from the HAP in terms of measuring health and wellbeing *in the retirement transition*?

## Recruitment and sampling

Semi-structured interviews will be conducted with a sample of participants in the trial from the intervention and control groups and two ‘reduced participation’ groups. The reduced participation groups will include people who initially declined to participate in the trial or to be randomised, but consented to a brief qualitative interview (n= up to 2), and people who withdraw from the trial but consent to a follow-up qualitative interview (n= up to 2), The number of participants interviewed from each group within the pilot trial is shown in Figure 3. In total therefore up to 28 participants will be included in the qualitative component of the pilot study, although the length of interview will vary by trial arm ranging from 60 to 20 minutes per interview. Interviews in the reduced participation groups may be considerably shorter (e.g. 10 minutes) in some instances.

|  | LEAP | Control | Reduced Participation |
| --- | --- | --- | --- |
| Participation stage: near baseline | (n≤ 8) | (n≤ 4) | Prior to randomisation: (n≤ 2) |
| Participation stage: near final assessment | (n≤ 8) | (n≤ 4) | Post randomisation: (n≤ 2) |
| Total: (n≤28) | **(n≤ 16)** | **(n≤ 8)** | **(n≤ 4)** |

**Fig 3. Diagram of the number of participants to be interviewed from each intervention group**

## Sampling procedure

The research questions cited above form the basis for our recruitment criteria. We will purposively sample from participants in the trial, aiming to achieve maximum variation with respect to our primary criteria outlined below.

Primary criteria: recruitment pathway (assuming assessment of different recruitment strategies); trial arm (2x conditions); gender; retirement stage (pre-retirement, partly retired, retired), occupation type (manual/non manual), socio-economic status.

Secondary criteria will shape sampling as the analysis develops (e.g. if gaps are identified in our qualitative dataset). Whilst it is not possible to identify these criteria at the outset, we anticipate that it will be necessary to recruit some participants in the following scenarios: module focus (e.g. if very few participants use a particular module, we will select those that do use the module to understand its attractiveness and function and the conditions which make it useful); participation level (fully participate, consent to qualitative study only, or incomplete participation).

## Data collection and analysis

Qualitative data will be collected through individual semi-structured interviews which will take place at the participants’ home or at a nearby location suitable for them. The scope and depth of the interviews will vary across groups. Participants drawn from the groups receiving the LEAP intervention will take part in an interview lasting up to 60 minutes. The interview schedule for this group is presented in Appendix B. Participants in the control condition will take part in an interview of up to 40 minutes duration. The interview schedule for this group is presented in Appendix C. Participants who initially declined to take part in the pilot, or those who withdrew from the pilot will take part in a brief interview of up to 20 minutes duration. Interview schedules for these groups are presented in Appendices D and E respectively.

We will conduct a thematic analysis (Guest etal., 2012) of the data using a coding manual developed by two of the research team (SM, BH) through preliminary analysis of data and the research questions and objectives. Data will be organised using a software package (NVivo).

## Anticipated time requirements

### Data collection

We anticipate approximately 6- 8 weeks for data collection (assuming all participants are already recruited to the trial and 4 interviews can be completed per week). In practice participants may take part in an qualitative interview on a rolling basis, following recruitment to the pilot study.

### Analysis

We estimate approximately 1 day to code a transcript (60-90 minute) using a basic coding frame. Therefore it will take approximately 6 weeks to initially code transcripts. It is difficult to estimate the time needed for analysis but for the purposes of writing a basic report we estimate approximately 5-6 weeks.

The total time to conduct the qualitative component of the pilot study therefore may take approximately 20 weeks.

# Data handling and record keeping

We will ask participants to read an information sheet about the study (Attachment 2 – Information sheet) and sign a consent form (Attachment 8 – consent form) prior to being recruited to the study.

Qualitative interviews (n=28) will be audio-recorded with respondents consent, and the recordings transcribed verbatim by an external transcription agency which have signed confidentiality agreements with Newcastle University. The single member of staff at the agency may be able to identify place and personal names from the audio-recordings. The transcripts will be anonymised during the process of transcription and will constitute data for analysis.

## Data Management

In line with good governance we will retain all primary data sources (audio recordings) for ten years so that they are available for re-analysis and audit should this requirement arise. Transcripts and audio recordings will be kept on a password protected secured server, accessible only to the research team throughout the duration of the LiveWell programme (current to 02/2015). Demographic information sheets and consent forms will be stored on university premises in a locked cabinet.

Permission for data sharing with third parties at the end of the study will be sought from all participants. All data to be shared will be rendered anonymous, and will be marked with a unique study identifier. For the first 24 months after the end of the LiveWell programme, the data will only be available to members of the LiveWell team (while further papers are being prepared for publication). Formal application and registration to use the data will be required. Anonymised transcripts of qualitative interviews will be offered in electronic form to the ESRC Qualidata Archive which manages preservation, access, and intellectual property aspects of data sharing.

# Ethics and regulatory issues

## LEAP intervention

The potential risks for participants using the LEAP intervention are minimal. The encouraged changes to physical activity level and dietary intake are positive and will be beneficial to all participants. The physical activity intervention promotes increasing daily physical activity through moving more and sitting less. The intervention does not promote high performance or elite exercise, where the risk of physical injury is high. The dietary intervention is intended to make changes towards a healthier eating style; it does not involve the testing of any food products, supplements or novel foods.

## HAP battery assessment

The HAP battery assessment comprises physical capability tests, questionnaires designed to assess psychological and social wellbeing, and cognitive function tests. Physical capability tests have been widely used and their compliance and acceptability is well known. These involve a minimum effort and so do not involve major risks to most participants. Qualitative process evaluation

The potential risks for participants in this research are minimal. However some participants may not wish to share some information about their personal circumstances, or provide information on the demographic data sheet. Some participants may feel uncomfortable discussing personal information within the interview. However, we will fully inform participants about the methods of data collection, and the aims and objectives of the research prior to their participation. In addition, we will take all reasonable steps to reassure participants that they should only provide information they are happy to divulge for the purposes of the research, that their information will be rendered anonymous, that participation is voluntary, that they may withdraw from the research at any time, and that they may request their data be removed from our records during the life of the study. We will also inform the participants that they may decide to withdraw themselves and their data from the qualitative process evaluation, but remain in the trial if they wish.

# Confidentiality

At the point of consent to the trial, all participants will be allocated an identification code. Any data from which the identity of the participant could be determined will be kept securely on a password protected secure university network accessible only to members of the research team (henceforth described as the ‘university network’), or in a locked filing cabinet in the Human Nutrition Research Centre (quantitative data). Data collected through the qualitative process evaluation will be stored by a research associate in a locked cupboard in the Institute of Health and Society or on the university network.

Qualitative interviews will be audio recorded using a digital recorder. The digital files (raw data) will be sent to an external transcription company who have signed a confidentiality agreement with Newcastle University. Digital files will be sent in encrypted format using TrueCrypt © software. The transcription company will not have any information about the participants other than that included in the digital recording. The person transcribing the focus group will remove any information that could identify participants from the transcript, replacing such information with the participants ID code. Transcripts will be returned to the university in an encrypted digital format and will be stored on the university network. Quotes from qualitative interviews will be used to illustrate emergent themes from the data but these will be coded to hide the identity of the participant. Data will be treated in a way that is ‘non-attributable.

# Participant reimbursement

Participants will each receive a voucher (Eldon square) to the value of £50 for their participation in the trial. This figure matches current INVOLVE (ref) recommendations for reimbursement for participants in research who are not taking research-design or leadership roles. The current recommendations are for participants to receive a minimum £10 per hour. To ensure ethical and methodologically sound research, participants randomised to different arms of the trial should all receive equal financial reimbursement, even though some participants may be required to invest more time in research activities (e.g. if randomised to the intervention rather than control arm). Estimated research time varies from approximately 4 hours (two HAP assessments) in the control arm through to 6 hours (2 HAP assessments and interaction with the intervention) in the intervention arm of the trial. Therefore 5 hours (£50 reimbursement) represents an average amount of time spent on research activities across all participants.

# Insurance and finance

# dissemination

## Conferences

The quantitative and qualitative outcomes of this pilot trial will be presented at national and international conferences attended by multidisciplinary and varying audiences, including the EHPS, UKSBM, ISBNPA etc.

## Publications

The quantitative and qualitative outcomes of this pilot trial will be written-up for publication in peer-reviewed journals. Target journals included …

## Stakeholder event

The outcomes of this pilot trial will be disseminated at a final stakeholder event anticipated to occur in…

# Projected Timeline to conduct Pilot RCT

| **LEAP Pilot Timeline** | | | | | |  |  |  |  |  |  |  |  |  |  |  |  |  |  |  |  |  |  |  |  |  |  |  |  |
| --- | --- | --- | --- | --- | --- | --- | --- | --- | --- | --- | --- | --- | --- | --- | --- | --- | --- | --- | --- | --- | --- | --- | --- | --- | --- | --- | --- | --- | --- |
|  |  |  |  |  |  |  |  |  |  |  | **2013** |  |  | **2014** |  |  |  |  |  |  |  |  |  |  |  | **2015** |  |  |  |
|  |  |  |  |  |  |  |  | **Months** | | | Oct | Nov | Dec | Jan | Feb | Mar | Apr | May | Jun | Jul | Aug | Sep | Oct | Nov | Dec | Jan | Feb | Mar |  |
| **LEAP Pilot RCT** | | | | |  |  |  |  |  |  |  |  |  |  |  |  |  |  |  |  |  |  |  |  |  |  |  |  |  |
| Protocol development | | | | | | |  |  |  |  |  |  |  |  |  |  |  |  |  |  |  |  |  |  |  |  |  |  |  |
| University ethics approval | | | | | | | |  |  |  |  |  |  |  |  |  |  |  |  |  |  |  |  |  |  |  |  |  |  |
| Participant recruitment | | | | | | |  |  |  |  |  |  |  |  |  |  |  |  |  |  |  |  |  |  |  |  |  |  |  |
| Baseline assessment | | | | | |  |  |  |  |  |  |  |  |  |  |  |  |  |  |  |  |  |  |  |  |  |  |  |  |
| 2 month follow-up assessment | | | | | | | | |  |  |  |  |  |  |  |  |  |  |  |  |  |  |  |  |  |  |  |  |  |
| Qualitative interviews | | | | | | |  |  |  |  |  |  |  |  |  |  |  |  |  |  |  |  |  |  |  |  |  |  |  |
| Data analysis and 'data clinics' | | | | | | | | |  |  |  |  |  |  |  |  |  |  |  |  |  |  |  |  |  |  |  |  |  |
| Write-up pilot trial outcomes | | | | | | | | |  |  |  |  |  |  |  |  |  |  |  |  |  |  |  |  |  |  |  |  |  |
| Develop protocol for a definitive RCT | | | | | | | | | | |  |  |  |  |  |  |  |  |  |  |  |  |  |  |  |  |  |  |  |
| Develop funding application for RCT | | | | | | | | | |  |  |  |  |  |  |  |  |  |  |  |  |  |  |  |  |  |  |  |  |

**References**

Bate SP, Robert G. Bringing user experience to health care improvement. Oxford: Radcliffe Publishing 2007.

Craig P, Dieppe P, Macintyre S, Michie S, Nazareth I, Petticrew M. Developing and evaluating complex interventions: the new Medical Research Council guidance. *British Medical Journal* 2008;337:a1655.

Guest G, MacQueen KM, Namey EE (2012). Applied thematic analysis. SAGE Publications, Inc Heaven, B., Brown, L. J., White, M., Errington, L., Mathers, J. C., & Moffatt, S. (2013). Supporting Well‐Being in Retirement through Meaningful Social Roles: Systematic Review of Intervention Studies. Milbank Quarterly, 91(2), 222-287.

**Hobbs N**, **Godfrey A**, **Lara** J, **Errington L**, **Meyer TD**, **Rochester L**, **White M**, **Mathers JC** and **Sniehotta FF (2013). Are behavioral interventions effective in increasing physical activity at 12 to 36 months in adults aged 55 to 70 years? a systematic review and meta-analysis.** BMC Medicine 2013, **11**:75. doi:10.1186/1741-7015-11-75.

Lancaster GA, Dodd S, Williamson PR. Design and analysis of pilot studies: recommendations for good practice. Journal of Evaluation in Clinical Practice. 2004;10(2):307-12.

Lara J, Hobbs N, Moynihan P, Meyer TD, Adamson AA, Errington L, Rochester L, Sniehotta FF, White M, Mathers JC. Effectiveness of interventions promoting components of a Mediterranean diet among adults of retirement age: a systematic of review and meta-analysis of randomised controlled trials. *Obesity Reviews (under review).*

Lara J, Godfrey A, Evans E, Heaven B, Brown LJE, Barron E, Rochester L, Meyer TD, Mathers JC. [Towards measurement of the Healthy Ageing Phenotype in lifestyle-based intervention studies](http://www.ncl.ac.uk/hnrc/research/publication/193746). *Maturitas* 2013, 76(2), 189-199.

Liu B, Young H, Crowe FL, Benson VS, Spencer EA, Key TJ, Appleby PN, Beral V. Development and evaluation of the Oxford WebQ, a low-cost, web-based method for assessment of previous 24 h dietary intakes in large-scale prospective studies. Public Health Nutr. 2011; 14: 1998-2005

Martınez-Gonzalez, M. A., Garcıa-Arellano, A., Toledo, E., Salas-Salvado, J., Buil-Cosiales, P., Corella, D., ... & n Estruch, R. (2012). A 14-Item Mediterranean Diet Assessment Tool and Obesity Indexes among High-Risk Subjects: The PREDIMED Trial. *PLoS One*, *7*, e43134.

Michie S, Ashford S, Sniehotta FF, Dombrowski SU, Bishop A, French DP. A refined taxonomy of behaviour change techniques to help people change their physical activity and healthy eating behaviours: The CALO-RE taxonomy. Psychology & Health. 2011 2011/11/01;26(11):1479-98.

NHS Institute for Innovation and Improvement. Experience based design: using patient and staff experiences to design better healthcare services. Coventry: NHS Institute for Innovation and Improvement 2009.

**APPENDIX A**

**HAP battery**

*Equipment*

The following equipment will be required to conduct the assessment:

Laptop computer

Blood pressure monitor and stethoscope MicroLife watchBPhome

Portable spirometer (and consumables) Micro 1 Handheld Spirometer. Carefusion.

Weighing scale. Tanita TBF 300-MA

Portable stadiometer

Hydraulic hand Dynamometer. Baseline evaluation Instruments

9-hole pegboard. Rolyan

Measuring tape

Marking tape

Chronometer

CANTAB Touch Screen Tablet computer

*Assessment domains*

**Physical activity and inactivity**

An activity monitor, Axivity, will be used to assess physical activity and sedentary behaviour over seven consecutive days. The thin, waterproof device is attached to the participant on the back between L4 and L5. The device collects data on bouts of sedentary and ambulatory activity. After seven days, the participant will remove the device themself and send it back to the study team using a stamped address prepaid envelope.

**Dietary intake**

Dietary intake will be assessed using the multiple pass 24-hour recall method. In order to assess usual food intake, before and after interventions participants will be asked to complete a 24-hour dietary recall on three days, including two weekdays and one weekend day. In order to reduce possible effects of between-day variability in food intake, participants will be required to complete these dietary recalls on the same days of the week at each timepoint (eg Thursday, Friday, and Saturday; or Sunday, Monday, and Tuesday). An online diet questionnaire, the Oxford WebQ (Liu et al 2011), will be used. The Oxford webQ questionnaire takes around 15 to 20 minutes to complete. The Oxford WebQ is self-administered and assesses what participants ate and drank the previous day.

Five ageing-related domains will be will be evaluated in this study, physiological and metabolic health, physical capability, cognitive function, psychological wellbeing, and social wellbeing. The procedure for administration of the Health Ageing Phenotype (HAP) is explained in the HAP administration guidelines (appendix Z).

1. Physiological and Metabolic Health Domain

a) Blood pressure. Resting blood pressure will be measured in triplicate using a manual BP monitor. Before the measurement participants will be invited to rest in a sitting position for at least 10 minutes. An appropriate cuff size will be used for the measurement of each subject which will be used for all other measurements of BP.

b) Forced expiratory volume (FEV1). FEV1 will be assessed before and after interventions with a portable handheld spirometer (Micro 1). Briefly the participant will take in as deep breath as possible, when full place the mouthpiece in his/her mouth. Close his/her lips tightly around the mouthpiece. Blow through the mouthpiece into the spirometer, blowing air out as hard, fast, smoothly and completely as possible. Record the FEV1 from at least two and up to five technically satisfactory manoeuvres.

c) Waist and hip circumferences. Waist and hip circumferences will be measured using a measuring tape and following standardised protocols. Waist circumference will be measured midway between the uppermost border of the iliac crest and the lower border of the costal margin (rib cage). The tape should be placed around the abdomen at the level of this midway point and a reading taken when the tape is snug but does not compress the skin. In practice it may be difficult for very overweight patients to accurately palpate those bony landmarks in which case placing the tape at the level of the belly button is recommended. For hip circumference, identify the widest part of the buttocks. Then place the tape measure at this location and measure around the circumference of hips and buttocks.

d)Body weight and height. Body weight and height will be measured without shoes, after removing heavy garments and emptying pockets, using a portable scale and stadiometer. Waist to hip ratio and body mass index (BMI) weight/(height)^2^ will be estimated from these anthropometric measures.

2. Physical Capability Domain (Reuben 2013)

a) Handgrip strength. Hand-grip strength will be assessed in both arms at baseline and after intervention to measure the maximum isometric strength of the hand and forearm muscles. A lightweight and portable dynamometer will be used. The researcher will follow a specific protocol for the measurement of hand-grip strength (i.e. allowing one practice trial, and then record the best of three attempts with 30 seconds rest between each of these). The test takes approximately 3 minutes to administer

b) Gait speed. The 4-Meter Walk Gait Speed will be used as a measure of locomotion. Participants are asked to walk a short distance (4 meters) at their usual pace. Participants complete one practice and then two timed trials. Raw scores are recorded as the time in seconds required to walk 4 meters on each of the two trials, with the better trial used for scoring. The test takes approximately 3 minutes to administer (including instructions and practice).

c) Walk endurance test. The 2-Minute Walk Endurance Test is used as the measure of endurance. This test measures sub-maximal cardiovascular endurance by recording the distance that the participant is able to walk on a course in 2 minutes. The participant’s raw score is the distance in feet and inches walked in 2 minutes. The test overall takes approximately 4 minutes to administer (with instructions and practice).

d) Dexterity. A 9-Hole Pegboard Dexterity Test is used as a measure of dexterity. This simple test of manual dexterity records the time required for the participant to accurately place and remove 9 plastic pegs into a plastic pegboard. The protocol includes 1 practice and 1 timed trial with each hand. Raw scores are recorded as time in seconds that it takes the participant to complete the task with each hand (a separate score for each). The test takes approximately 4 minutes.

e) Standing balance test. The Standing Balance Test is a measure developed to assess static standing balance. It involves the participant assuming and maintaining up to 5 poses for 50 seconds each. The sequence of poses is: eyes open on a solid surface, eyes closed on solid surface, eyes open on foam surface, eyes closed on foam surface, eyes open in tandem stance. This test takes approximately 7 minutes to administer.

f) Sit-to-stand test. Participants start seated and then are timed standing up and sitting back down a 5 times and time will be recorded. Participants are usually encouraged to complete the test as fast as they can and are told to keep their arms folded. Participants will do one practice plus 2 timed trials.

g) Timed-up-and-go (TUG). Subjects asked to stand up from a standard chair with a seat height of between 40 and 50 cm, walk a 3-m distance at a normal pace, turn, walk back to the chair, and sit down. Timing measured in seconds began at the word ‘‘go’’ and ended when the subject’s back touched the backrest of the chair. Participants will perform three trials.

3. Cognitive Function

a) Executive function. Verbal and category fluency tests will be used to assess participants’ executive function. Within a set timeframe, usually one minute per letter participants are instructed by the experimenter to speak aloud as many words as possible that begin with a specific letter (phonemic fluency) – this is repeated 3 times for 3 different letters (e.g., CFL or PRW). In a separate one minute window, participants are then instructed to speak aloud as many words as possible which belong to a specific category, such as animals (category fluency). Participants will be audio-recorded when performing these tasks, to allow for detailed analyses of the responses made, including total number of words, repetition and errors. Completion of these tests takes approximately 5 to 7 minutes.

b) Episodic memory. The paired associates learning (PAL) CANTAB test will be used. Boxes are displayed on a touchpad screen. The participant watches as the boxes are ‘opened’ automatically, one by one, in a randomised order. One or more of them will contain a pattern. The patterns are then displayed in the middle of the screen, one at a time, and the participant must touch the box where the pattern was originally located. If the participant makes an error, the patterns are re-presented to remind the participant of their locations. The difficulty level increases through the test. The number of patterns increases from one (with 7 empty boxes) to eight (with no empty boxes), which challenges even very able participants.

Test administration takes approximately 10 minutes, depending on level attained. The PAL has 21 outcome variables including errors made by the participant, the number of trials required to locate the pattern(s) correctly, memory scores and stages completed. These data are produced automatically.

c) Processing speed. The letter-digit substitution test will be used to assess participants’ speed of processing. Participants complete this test using a pen and paper, upon which is presented a test grid and a key. The test grid is partially completed: a series of letters fills the top row of the grid, whilst the bottom row is blank. The key gives the numbers 1 to 9, each of which is paired with a different letter. Participants are required to fill in the blank boxes below the randomized letters with the appropriate digit indicated by the key, either by writing the digit in the response sheet or by speaking the digit aloud. The first 10 items are used as practice items, to ensure that participants understand the test instructions. After completion of these items, participants are instructed to replace the remaining items as quickly as possible. The key and the stimuli are the same for the oral and written versions of the test. Administration takes approximately 5 minutes. The number of correct substitutions made in 60 seconds is the dependent variable for both test versions. Responses can be given verbally or in writing (norms available for both), accommodating participants with motor limitations.

4. Psychological Wellbeing

a) Positive affect. The NIH Toolbox Positive Affect Survey will be used to assess participants’ levels of positive affect. This tool is administered via the NIH Assessment Center website. Items, displayed on a computer screen, ask about happiness, serenity, and cognitive engagement. The participant is asked to indicate agreement with each item using a 5 point Likert scale ranging from “not at all” to “very much”. Administration of this test takes 3-5 minutes; scoring and administration are automated. Computer adaptive testing is used in order to avoid practice effects

b) Life satisfaction. The satisfaction with life scale (SWLS) will be used. This tool is administered via the NIH Assessment Center website. Participants are prompted to rate 5 statements using a 7-point Likert scale to indicate the extent to which they agree with each statement (strongly agree to strongly disagree). The total is calculated automatically by the Assessment Center. Higher scores indicate greater satisfaction and administration of this test takes 1-2 minutes.

c) Quality of life. The control, autonomy, pleasure and self-realization, quality of life scale (CASP-19) will be used to assess participants’ multidimensional quality of life. This tool is administered via the NIH Assessment Center website. The test consists of 19 items relating to four theoretically-derived domains: Control (4 items), Autonomy (5 items), Pleasure (5 items), Self-realisation (5 items). Participants report the frequency with which they experience certain feelings or cognitions using a four-point Likert scale ranging from “Often” to “Never.” Items are scored automatically via the Assessment Center (with reverse coding of positive responses, so that higher scores equal higher QoL. The authors define the scale ranges as 0 (complete absence of QoL) to 57 (total satisfaction in all four domains). Administration of the test takes ~ 5 minutes

d) Mental health. The centre for epidemiological studies depression scale (CES-D) will be used to assess participants’ mental health, with a specific focus on depressive symptomatology. This test is administered via the NIH Assessment Centre. Participants are prompted to respond to 20 items by indicating the frequency with which they have experienced depressed mood, feelings of guilt, worthlessness and helplessness, psychomotor retardation, loss of appetite and sleep difficulties over the previous 7 days. Responses range from often (5 or more days out of 7) to not at all (one or zero days). Completion of the test takes approximately 5 minutes and the score is calculated automatically by the NIH Assessment Center.

5. Social Wellbeing (Gershon et al., 2010, Salsman et al., 2013)

We intend to use the following measurement tools, all of which will be administered via the National Institute of Health (NIH) Assessment Centre though a computer or smartphone and internet connection. The instruments can also be administered in paper and pencil format as short form instruments (typically from six to 14 items), although an inclusion criteria to the trial is that participants have access to the internet.

There is evidence that provision of explicit social roles (occupations) with group support can produce increased health and wellbeing in retirement (Heaven et al, 2013). However, a suitable instrument that assesses the perceived meaningfulness and satisfaction with explicit roles has not been identified. Consequently for use in the proposed pilot study we will use two versions (pre and post exposure) of a brief questionnaire developed by the research team. The questionnaire is currently untested and there is no psychometric information available regarding its properties. However, given the lack of appropriate measurement tools in this domain, it will collect self-reported expectation and (post intervention experience) of engaging in a social role/occupation. Currently the questionnaire is composed of 11 items in both (pre and post) versions and can be delivered online via the NIH Assessment Centre.

A link will be provided to the social relationships assessment tools at the NIH Assessment Centre. On following the link participants will be guided by text-based instruction through the assessment instruments. This will involve registering with the assessment centre (providing gender, and date of birth) and then entering an identification code and a password generated by the assessment centre. The participant will then follow onscreen instructions to complete the instruments which are presented sequentially will be included alongside measures of social wellbeing. Completion of both psychological and social wellbeing instruments takes approximately 8-10 minutes. The specific instruments are:

a) (PROMIS) companionship and social isolation scales: assesses perceived availability of someone with whom to share enjoyable social activities such as visiting, talking, celebrations, etc. It comprises 6 items, each with a scale 1-5 (Never to always). The PROMIS Social Isolation item bank assesses perceptions of being avoided, excluded, detached, disconnected from, or unknown by, others. This scale comprises 14 items, each with a scale 1-5 (Never to always).

b) PROMIS satisfaction with social roles scale: measures satisfaction with capacity to engage socially with family and friends and to perform social responsibilities. In total 44 items are available, however the respondent would only complete as many items as necessary in each bank to reach a level of internal consistency using Computerised Adaptive Testing (CAT).

c) PROMIS emotional and informational support bank: The PROMIS Emotional Support item bank assesses perceived feelings of being cared for and valued as a person and having confidant relationships. The PROMIS Informational Support item bank assesses perceived availability of helpful information or advice.

d) NIH toolbox meaning and purpose inventory: Meaning and Purpose is characterized by the extent to which people feel their life matters or makes sense. This tool comprises a total of 18 items rated on a 5-point Likert scale from strongly agree to strongly disagree.

**APPENDIX B**

**LEAP Intervention Interview Schedule**

# Topic Guide: LEAP participants (early participation)

**Participation in the trial**

1. How did you come to be involved in this research? (explore recruitment pathway, materials etc.).
2. Why did you agree to take part in the study (what was attractive/interesting)?
3. What are your expectations for the study? (what do you hope to get from it?).
4. How have you found being in the study so far (what has surprised you/been as expected?).
5. How do you feel about having been chosen by chance to get access LEAP? (explore concept of randomisation – do participants understand it)?

**Experience with LEAP**

Function

1. What are your general impressions of LEAP?
   1. What did you think of LEAP when you first saw it?
2. Talk me through how you have used LEAP so far (use site map document to support discussion about pathway through website)
   1. What are the reasons for using particular modules and tools but not others?
   2. What is useful about each module and tool, and what is not useful? (what could be improved?)
   3. What problems did you have in using LEAP?
   4. What worked well?
   5. Have you used any other sources of information outside LEAP? (e.g. other websites)

1. What was your experience of the following features (did they make sense, were they useful?):
   1. Dashboard?
   2. Diary (i.e. activity planner)?
   3. Notifications and prompts?
   4. The mentor?

*Concept & expectation*

1. In what ways has LEAP been what you expected?
   1. How has it been different?
2. What do you expect will happen at the end of the study:
   1. To how you live? (will LEAP change your behaviour)?
   2. To your use of LEAP (would you continue to use it if it was available)?
3. Would you recommend LEAP to another person (if so why)?
   1. Have you shared your log-in details with anyone else?
4. Is there anything important we haven’t discussed about your experience of LEAP?

**Experience with HAP**

You took part in some measures of your health (HAP).

1. What was your experience of:
   1. Time taken
   2. The number/range of measures (did it feel comprehensive or too much)?
   3. What was difficult to do (if anything)
2. If you didn’t have the measures, would you have used LEAP differently?

# Topic Guide: LEAP participants (late participation)

**Participation in the trial**

1. How did you come to be involved in this research? (explore recruitment pathway, materials etc.).
2. Why did you agree to take part in the study (what was attractive/interesting)?
3. What are your expectations for the study? (what do you hope to get from it?).
4. How have you found being in the study (what has surprised you/been as expected?).
5. Looking back - what factors helped you to stay to the end?
6. Were there any things that tempted to you leave the study? (was anything difficult or taxing?)
7. How did you feel about having been *chosen by chance* to get access LEAP? (explore concept of randomisation – do participants understand it)?

**Experience with LEAP**

*Function*

What are your general impressions of LEAP?

- 1. What did you think of LEAP when you first saw it?

1. Talk me through how you have used LEAP (use site map document to support discussion about pathway through website)
   1. What are the reasons for using particular modules and tools but not others?
   2. What is useful about each module and tool, and what is not useful? (what could be improved?)
   3. What problems did you have in using LEAP?
   4. What worked well?
   5. Did you use any other sources of information outside LEAP? (e.g. other websites)

1. What was your experience of the following features (did they make sense, were they useful?):
   1. Dashboard?
   2. Diary (i.e. activity planner)? – have participants continued to use this?
   3. Notifications and prompts?
   4. The mentor?

*Concept & expectation*

1. In what ways has LEAP been what you expected?
   1. How has it been different?
2. What do you expect will happen at the end of the study:
   1. To how you live? (will LEAP change your behaviour)?
   2. To your use of LEAP (would you continue to use it if it was available)?
3. Overall how would you describe LEAP (i.e., engaging, fun, boring, easy to use, intuitive, innovative etc.?)
   1. Would you recommend LEAP to another person (if so why)?
   2. Have you shared your log-in details with anyone else?
4. Is there anything important we haven’t discussed about your experience of LEAP?

**Experience with HAP**

You took part in some measures of your health (HAP).

1. What was your experience of:

a. Time taken

b. The number/range of measures (did it feel comprehensive or too much)?

c. What was difficult to do (if anything)

2. If you didn’t have the measures, would you have used LEAP differently?

**APPENDIX C**

**Control Group Interview Schedule**

**Topic Guide: NHS ‘LiveWell’ participants (early participation)**

**Participation in the trial**

1. How did you come to be involved in this research? (explore recruitment pathway, materials etc.).
2. Why did you agree to take part in the study (what was attractive/interesting)?
3. What are your expectations for the study? (what do you hope to get from it?).
4. How have you found being in the study so far (what has surprised you/been as expected?).
5. How do you feel about having been chosen by chance to get access the NHS LiveWell website? (explore concept of randomisation – do participants understand it)?

**Experience with NHS ‘LiveWell’ site**

Function

1. What are your general impressions of the NHS website?
   1. What did you think of website when you first saw it?
2. Talk me through how you have used the NHS ‘LiveWell’ site
   1. What parts of the site did you find most helpful? (which parts were least useful)?
3. In what ways has NHS ‘LiveWell’ been what you expected?
   1. How has it been different?
   2. Have you used any other sources of information outside LiveWell? (e.g. other websites)
4. What do you expect will happen at the end of the study:
   1. To how you live? (will the information gain from the website change your behaviour)?

b. To your use of the website (would you continue to use it if it was available)?

5. Would you recommend NHS LiveWell to another person (if so why)?

1. Is there anything important we haven’t discussed about your experience of the website?

**Experience with HAP**

You took part in some measures of your health (HAP).

1. What was your experience of:
   1. Time taken
   2. The number/range of measures (did it feel comprehensive or too much)?
   3. What was difficult to do (if anything)
2. If you didn’t have the measures, would you have used LEAP differently?

**Topic Guide: NHS ‘LiveWell’ participants (late participation)**

**Participation in the trial**

1. How did you come to be involved in this research? (explore recruitment pathway, materials etc.).
2. Why did you agree to take part in the study (what was attractive/interesting)?
3. What are your expectations for the study? (what do you hope to get from it?).
4. How have you found being in the study (what has surprised you/been as expected?).
5. Looking back - what factors helped you to stay to the end?
6. Were there any things that tempted to you leave the study? (Was anything difficult or taxing?)
7. How did you feel about having been *chosen by chance* to get access to the NHS LiveWell website? (explore concept of randomisation – do participants understand it)?

**Experience with NHS ‘LiveWell’ site**

Function

1. What are your general impressions of the NHS website?
   1. What did you think of website when you first saw it?
2. Talk me through how you have used the NHS ‘LiveWell’ site
   1. What parts of the site did you find most helpful? (which parts were least useful)?
3. In what ways has NHS ‘LiveWell’ been what you expected?
   1. How has it been different?
   2. Have you used any other sources of information outside LiveWell? (e.g. other websites)
4. What do you expect will happen at the end of the study:
   1. To how you live? (will the information gain from the website change your behaviour)?

b. To your use of the website (would you continue to use it if it was available)?

5. Would you recommend NHS LiveWell to another person (if so why)?

1. Is there anything important we haven’t discussed about your experience of the website?

**Experience with HAP**

You took part in some measures of your health (HAP).

1. What was your experience of:
   1. Time taken
   2. The number/range of measures (did it feel comprehensive or too much)?
   3. What was difficult to do (if anything)
2. If you didn’t have the measures, would you have used LEAP differently?

**APPENDIX D**

**Declined Participation Interview Schedule**

**TOPIC GUIDE: ‘NON-PARTICIPANTS’**

1. How did you come to be involved in this research? (explore recruitment pathway, materials etc.).
2. Why did you initially agree to take part in the study (what was attractive/interesting)?
3. What were your expectations for the study? (what do you hope to get from it?).
4. Please tell me how you came to leave the study? (explore reasons for leaving)
5. You’ve kindly agreed to this interview: what prompted you to take part in this discussion?
6. What factors might have encouraged you to stay in the trial had they been available at the time?
7. How did you feel about having been chosen by chance to [condition)?
8. Is there anything else we haven’t discussed about your participation in the study?

**APPENDIX E**

**Withdrew Participation Interview Schedule**

**TOPIC GUIDE: ‘NON-PARTICIPANTS’**

1. How did you come to be involved in this research? (explore recruitment pathway, materials etc.).
2. Why did you initially agree to take part in the study (what was attractive/interesting)?
3. What were your expectations for the study? (what do you hope to get from it?).
4. Please tell me how you came to leave the study? (explore reasons for leaving)
5. You’ve kindly agreed to this interview: what prompted you to take part in this discussion?
6. What factors might have encouraged you to stay in the trial had they been available at the time?
7. How did you feel about having been chosen by chance to [condition)?
8. Is there anything else we haven’t discussed about your participation in the study?

**References for Appendix**

Reuben DB, Magasi S, McCreath HE, Bohannon RW, Wang YC, Bubela DJ, Rymer WZ, Beaumont J, Rine RM, Lai JS, Gershon RC. Motor assessment using the NIH Toolbox. Neurology. 2013; 80(11 Suppl 3): S65-75.

Robbins TW, James M, Owen AM, Sahakian BJ, McInnes L, Rabbitt P. Cambridge Neuropsychological Test Automated Battery (CANTAB): a factor analytic study of a large sample of normal elderly volunteers. Dementia, 5 (1994), pp. 266–281

Jolles, J., Houx, P.J., Van Boxtel, M.P.J. and Ponds, R.W.H.M. 1995. Maastricht Aging Study: Determinants of cognitive aging, Maastricht, The Netherlands: Neuropsych Publishers.

Diener E, Emmons RA, Larsen RJ, Berkman LF. The satisfaction with life scale. Journal of Personality Assessment. 1985; 49(1): 71–5.

Hyde M, Wiggins RD, Higgs P, Blane DB. A measure of quality of life in early old age: the theory, development and properties of a needs satisfaction model (CASP-19). Aging and Mental Health. 2003; 7: 186–194.

Radloff LS (1977) The CES-D scale: A self-report depression scale for research in the general population. Appl Psychol Meas 1: 385–401.

Gershon, R.C., Rothrock, N., Hanrahan, R., Bass, M. & Cella, D. (2010). The use of PROMIS and Assessment Center to deliver patient-reported outcome measures in clinical research. Journal of Applied Measurement, 11, 304-314.

Salsman JM, Butt Z, Pilkonis PA, Cyranowski JM, Zill N, Hendrie HC, Kupst MJ, Kelly MA, Bode RK, Choi SW, Lai JS, Griffith JW, Stoney CM, Brouwers P, Knox SS, Cella D. Emotion assessment using the NIH Toolbox. Neurology. 2013;80(11 Suppl 3):S76-86
